# Ultra-sensitive glyphosate detection in soil and wastewater using Zn-zeolitic imidazolate framework-67/montmorillonite nanocomposite electrochemical sensor

**DOI:** 10.1007/s00604-026-07880-4

**Published:** 2026-03-11

**Authors:** Mona Elfiky, Amr M. Beltagi, Marwa M. Bediwy

**Affiliations:** 1 Chemistry Department, Faculty of Science, Tanta University, Tanta, 31527 Egypt; 2 Department of Chemistry, Faculty of Science, Kafrelsheikh University, Kafrelsheikh, 33516 Egypt

**Keywords:** Glyphosate herbicide, Zinc-based zeolitic imidazolate framework-67, Exfoliated montmorillonite, Anodic stripping voltammetry, Environmental monitoring

## Abstract

A novel electrochemical sensor for the determination of glyphosate (GLY) has been developed. It is based on a nanocomposite of zinc-based zeolitic imidazolate framework-67 and exfoliated montmorillonite (Zn-ZIF-67/Exf. MMt). The composite was synthesized by a hydrothermal method and characterized using Fourier transform infrared spectroscopy (FT-IR), X-ray powder diffraction (XRD), transmission electron microscopy (TEM), and surface area analysis (BET). Incorporated into a modified graphite paste sensor (MGPS), the material provided high conductivity, large electroactive surface area (0.68 cm^2^), and low resistivity (75.0 Ω). Using square wave adsorptive cathodic stripping voltammetry (SW-AdCSV), the optimized sensor, containing 1.0% (Zn-ZIF-67/0.5 Exf. MMt) modified graphite paste sensor (MGPS), showed excellent electrochemical performance with a high electroactive surface area (0.68 cm^2^), and low resistivity (75.0 Ω). Under standard conditions, the sensor achieved a low detection limit of 0.009 nM (linearity range: 0.03-1.0 nM), and 0.015 nM (linearity range: 0.05–1.2 nM) in bulk, and spiked soil, respectively. This sensor offers a practical and highly sensitive tool for early detection of glyphosate, supporting environmental safety and sustainable agriculture.

**Graphical Abstract:**

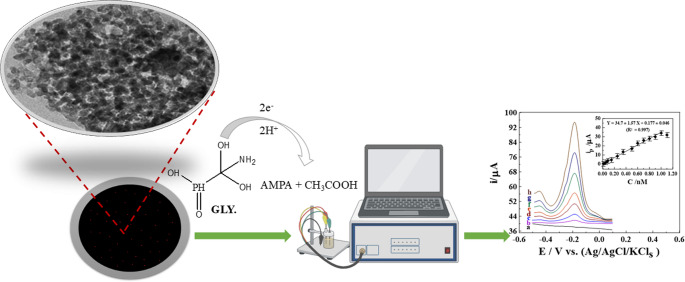

**Supplementary Information:**

The online version contains supplementary material available at 10.1007/s00604-026-07880-4.

## Introduction

**Scheme 1:**
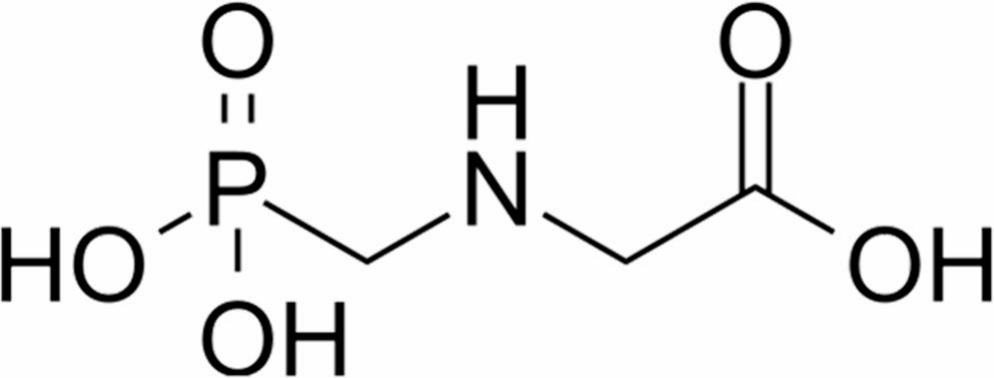
Structure of glyphosate (N-(phosphonomethyl)glycine, GLY)

Herbicides have become integral to modern agricultural practices globally. Nevertheless, despite their efficacy in pest management, these chemicals pose considerable hazards to natural ecosystems [1]. Glyphosate (N-(phosphonomethyl)glycine, GLY) [Scheme 1] is a widely used non-selective herbicide, representing approximately 60–72% of global pesticide consumption [2]. It is applied across agricultural, urban, and domestic environments, leading to significant contamination of water, soil, and food. Due to its persistence, GLY often infiltrates groundwater and drinking water sources, resulting in broad human exposure. Regulatory limits vary considerably: the U.S. Environmental Protection Agency allows up to 700 µg/L (~ 4.1 µM) in drinking water, whereas the European Union sets a much lower threshold of 0.1 µg/L (~ 0.6 nM) [3, 4]. Maximum residue levels (MRLs) also differ, with Canada and the U.S. permitting 1.66 and 4.14 µM, respectively [5]. Initially introduced by Monsanto in 1974 as Roundup^®^ and considered low in toxicity, GLY was reclassified in 2015 by the International Agency for Research on Cancer as a probable human carcinogen. Long-term exposure has been associated with endocrine disruption, developmental abnormalities in experimental models, and damage to multiple organ systems, including the nervous, cardiovascular, reproductive, hepatic, and renal systems, along with skin and gastrointestinal irritation [6–8]. GLY is difficult to detect and quantify due to its low volatility, high water solubility, and absence of chromophoric groups [9].

Various analytical methods have been developed, including chromatography [10, 11], spectroscopy [12–14], nuclear magnetic resonance [15], and mass spectrometry [16]. These techniques often require separation steps, such as gas or liquid chromatography, and derivatization to enhance selectivity and sensitivity at low concentrations [17]. However, they are expensive, time-consuming, and demand skilled personnel, making them unsuitable for routine on-site analysis. Conversely, electrochemical sensors have considerable advantages compared to conventional laboratory techniques such as Chromatography and spectroscopy, principally due to their appropriateness for on-site, real-time, economical, and portable analysis [18–22]. Therefore, several electrochemical sensors have been developed as promising alternatives for GLY detection [23]. Detection is typically achieved through GLY-induced changes in electrochemical signals, either by inhibiting biochemical reactions or binding to specific biomolecules such as enzymes, aptamers, or antibodies, allowing for label-free quantification [23, 24]. A wide range of electrochemical and optical sensors has been developed for GLY detection in environmental and food samples, demonstrating significant advances in sensitivity, selectivity, and matrix compatibility. Techniques such as cyclic voltammetry (CV), differential pulse voltammetry (DPV), square wave adsorptive cathodic stripping voltammetry (SW-AdCSV), and surface-enhanced Raman spectroscopy (SERS) have been employed across various sensor platforms, as listed in [Table 1]. For instance, the MWCNT/CuNP/Py-modified GCE sensor achieved a low detection limit of 0.002 µM with a linear range of 0.01–1.0 µM in soil and vegetable samples [25], while the silane-modified smectite/CPE sensor showed a detection limit of 0.98 µM in contaminated soil [26]. Sensors based on carbon black/NbNP-MSPE [27], porous biochar/nZVI-MS [28], and ZnO-NPs/PDDA-modified SPAgE [29] demonstrated effective performance in water and juice matrices, with detection limits ranging from 2.84 to 3.07 µM. SERS-based platforms such as rGO/AgNPs/TiO_2_ NTs achieved nanomolar sensitivity in water and soil [30], and ECL/amperometric sensors using nano-ZnO-decorated MWCNT or Au SPE reported detection below 1 µM [31]. The WaveFlex biosensor incorporating AuNPs/ZnO-NWs/MoS_2_-NSs reached 3.53 µM in soybean and corn samples [32], while a simple Au sensor detected glyphosate at 2 µM in tap water [33]. Green ZnO NP-modified SPE offered broad linearity (0.5 µM–7.5 mM) and detection limits of 0.648 µM and 0.96 µM in laboratory and river water, respectively [34].

**Table 1:** Summary of electroanalytical sensor platform and key performance metrics for GLY detection

Sensor Name	Tech.	LR	LOD	Matrix		Ref.
MWCNT/CuNP/Py-modified GCE	CV & DPV	0.01–1.0 µM	0.002 µM	Soil, spinach, peas, carrots, tomatoes	[25]	
Silane-modified smectite/CPE	CV	10–100 µM	0.98 µM	Contaminated soil	[26]	
Carbon black/NbNP-MSPE	DPV	5.90–172.30 µM	3.07 µM	Water	[27]	
Porous biochar/nZVI-MS	CV, LSV, DPV	Not specified	0.13 ppm (~ 769 nM)	Milk, apple juice, drinking water	[28]	
ZnO-NPs/PDDA-modified SPAgE	DPV	0–5 mM	2.84 µM	Green tea, corn juice, mango juice	[29]	
rGO/AgNPs/TiO₂ NTs SERS substrate	SERS	Not specified	3 µg/L (~ 17.74 nM)	Water, soil	[30]	
Nano-ZnO decorated MWCNT or Au SPE	ECL, amperometry	Not specified	< 1 µM	Water	[31]	
Wave Flex biosensor (AuNPs/ZnO-NWs/MoS₂-NSs)	LSPR	0–80 µM	3.53 µM	Soybean, corn	[32]	
Au sensor	Amperometry	Not specified	2 µM	Tap water	[33]	
Green ZnO NP-modified SPE	DPV	0.5 µM-7.5 mM	0.648 µM(lab) 0.96 µM (river)	River water	[34]	
Zn-ZIF-67/0.5 Exf. MMt MGPS	SW-AdCSV	0.03–1.0 nM 0.05–1.2 nM - -	0.009 nM 0.015 nM - -	Bulk Soil Brackish water Wastewater	**This study**	

Metal-organic frameworks (MOFs) [35] are emerging porous materials known for their high adsorption capacity and well-defined pore structures [36]. Despite these advantages, many MOFs suffer from poor electrical conductivity and limited stability, which restrict their use in electrochemical sensing. Zeolitic imidazolate frameworks (ZIFs) represent a subclass of MOFs [37], typically synthesized through self-assembly of metal ions such as Zn^2+^ or Co^2+^ with imidazole-based ligands. ZIFs offer excellent thermal and hydrothermal stability, large surface area, and strong adsorption properties, making them suitable for various applications [38]. Among them, ZIF-67 features a rhombic dodecahedral morphology with a three-dimensional porous structure [38]. Its cobalt content provides redox activity, while its structural stability and porosity enhance its performance as a sensing material [38, 39]. To overcome the limited conductivity of ZIF-67, multi-metallic ZIFs have been developed by combining different metal ions, which can improve electrochemical performance and sensing efficiency [40, 41].

Montmorillonite (MMt) is the most common smectite clay mineral, characterized by a layered hydrous aluminosilicate structure. Its lamellae are irregularly arranged, typically measuring ~ 100 nm in diameter and ~ 1 nm in thickness. Structurally, MMt consists of two tetrahedral silica sheets fused to a central octahedral aluminum hydroxide layer. The interlayer spaces contain exchangeable cations (e.g., Na^+^ in sodium-MMt) and water molecules, which help neutralize the negative charge generated by isomorphic substitution within the layers. This feature enables MMt to be modified and intercalated with various guest species, including metal oxides [42], MOFs [43, 44], and conducting polymers [45], forming hybrid or nanocomposite materials with enhanced physical and chemical properties for diverse applications. Despite these developments, no detailed study has yet explored the fabrication of nanocomposites based on porous Zn-ZIF-67 combined with various contents of exfoliated MMt nanosheets (Zn-ZIF-67/Ex.MMt) for electrochemical sensing.

In this study, a porous Zn-ZIF-67/Ex.MMt nanocomposite was synthesized via a solvothermal method using Zinc (II) nitrate, cobalt (II) nitrate, and 2-methylimidazole (2-MIM) as organic linker, with varying amounts of MMt nanosheet. The resulting material was employed to fabricate a highly sensitive GPS for the electrochemical detection of GLY in real agricultural wastewater, brackish water, and soil samples, utilizing square wave adsorptive cathodic stripping voltammetry (SW-AdCSV).

## Experimental part

### Materials and characterization techniques

Sodium montmorillonite (Na-MMt) clay, sourced from Southern Clay Products (Colloid BP), Inc. (Gonzales, Texas, USA), possesses a cation exchange capacity of 114.8 meq/100 g. Prior to use, the clay was dried in a vacuum oven at 100 °C for 24 h, yielding an interlayer spacing (d_001_) of 9.6 Å. All chemicals were obtained from Sigma-Aldrich and used without further purification. These included cobalt(II) nitrate hexahydrate (Co(NO_3_)_2_·6H_2_O, ≥ 98%), zinc nitrate hexahydrate (Zn(NO_3_)_2_·6H_2_O, ≥ 98%), 2-methylimidazole (2-MIM, ≥ 99%), potassium hexacyanoferrate (II) (K_4_[Fe(CN)_6_], ≥ 99.0%), ethanol (EtOH, ≥ 98%), N, N-dimethylformamide (DMF, ≥ 98%), phosphoric acid (H_3_PO_4_, ≥ 99.0%), boric acid (H_3_BO_3_; ACS reagent, ≥ 99.5%), acetic acid (CH_3_COOH; glacial, ACS reagent, ≥ 99.7%), sodium chloride (NaCl), sodium phosphate monobasic (NaH_2_PO_4_, ≥ 99.0%), disodium hydrogen phosphate dihydrate (Na_2_HPO_4_·2H_2_O, ≥ 99.5%), trisodium phosphate (Na_3_PO_4_, ≥ 96.0%), sodium hydroxide pellets (NaOH, ≥ 98.0%), potassium chloride (KCl, ≥ 99.0%), and GLY standard solution (1000 µg/mL in H_2_O).

The morphology and surface features of the synthesized nanocomposites were analyzed using field emission scanning electron microscopy (FE-SEM, Quanta™ 250) and high-resolution transmission electron microscopy (HR-TEM, JEM-2100 JEOL) with carbon-coated copper grids (200 mesh). Fourier transform infrared (FT-IR) spectra were recorded using a PerkinElmer spectrophotometer, while X-ray diffraction (XRD) patterns were obtained using a Rigaku Ultima IV R185 diffractometer equipped with Cu-K_α_ radiation (λ = 1.54 Å), operated at 40 kV and 20 mA. Prior to surface area analysis, the nanocomposite was degassed under vacuum at 150 °C for 2 h. Specific surface area and pore size distribution were determined using the Brunauer–Emmett–Teller (BET) method. Electrochemical impedance spectroscopy (EIS) and stripping voltammetric measurements were performed using a Solarton SI-1287 potentiostat with a 1252 A response analyzer, and a PAR 263 A computer-controlled potentiostat, respectively.

### Preparation of electroanalytical solutions

A (0.001 M) GLY solution was prepared by diluting 16.93 mL of the 1000 µg/mL stock solution (~ 0.00591 M) with 83.07 mL of double-distilled water (DDW) to obtain 100 mL of solution. Subsequently, it was further diluted with DDW to obtain concentrations ranging from 10 µM to 0.001 µM. Britton–Robinson buffer solutions (BRB; pH 2–12) were prepared by mixing 0.04 M boric, phosphoric, and acetic acids with 0.2 M NaOH in suitable proportions. Phosphate-buffered saline (PBS) solutions within the same pH range were obtained by combining equal volumes of 0.1 M K_2_HPO_4_ and KH_2_PO_4_, with pH adjusted using 1 M HCl or NaOH. Additionally, a 0.1 M HCl solution was prepared. All buffer systems, including BRB, PBS, and HCl, were employed as supporting electrolytes in the electrochemical experiments. For redox probe analysis, a 1.0 mol/L stock solution of K_4_[Fe(CN)_6_] and a 0.1 mol/L solution of KCl were freshly prepared and utilized in the electrochemical characterization of the fabricated sensor. To simulate environmental conditions, 0.5 g of soil was spiked with known concentrations of GLY in a microtube containing 5.0 mL DDW and a suitable buffer solution. The mixture was sonicated for 30 min. and then employed for subsequent analytical evaluations.

### Synthesis of Zn-ZIF-67 framework, and Zn-ZIF-67/Exf. MMt nanocomposites

**Scheme 2:**
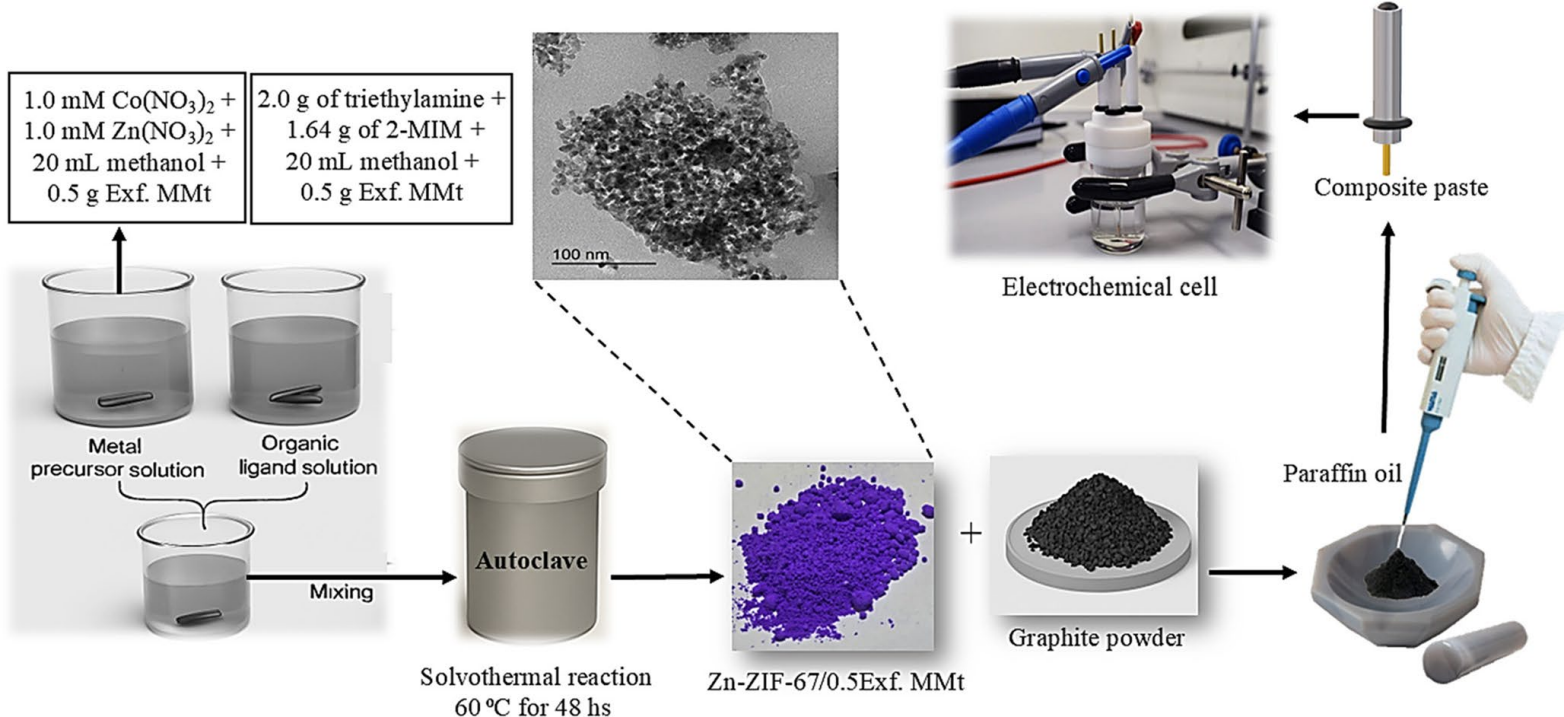
Schematic illustration of the synthesis and fabrication process of modified sensors

1 mM of Zn(NO_3_)_2_·6H_2_O and 1 mM of Co(NO_3_)_2_·6H_2_O were dissolved in 20 mL of methanol under continuous magnetic stirring to prepare the metal precursor solution (beaker A). In parallel, 2.0 g of triethylamine and 1.64 g of 2-MIM were dissolved in a separate 20 mL solution of methanol to formulate the organic ligand solution (beaker B) via solvothermal method [46]. The two solutions were subsequently mixed under stirring to facilitate the coordination-driven assembly of the framework. The resulting mixture was transferred into a Teflon-lined stainless-steel autoclave and subjected to solvothermal treatment at 60 °C for 48 hF in an oven, as illustrated in [Scheme 2]. The solid product was then recovered by centrifugation, thoroughly washed with methanol and subsequently dried in an oven at 80 °C for 12 h. Because of the tetrahedral coordinated cobalt, the synthesized sample exhibits a distinctive violet color as seen in [Scheme 2] [47]. A Zn-ZIF-67/0.5 Exf. MMt nanocomposite was synthesized using the same procedure, with the addition of 0.5 g of exfoliated Na-MMt clay in (beaker A) [44]. The same procedure was performed utilizing 1.0 g (1.0%), and 2.0 g (2.0%) of Exf. MMt clay to prepare Zn-ZIF-67/1.0 Exf. MMt, and Zn-ZIF-67/2.0 Exf. MMt nanocomposites, respectively.

### Fabrication of bare and modified sensors

As illustrated in [Scheme 2], to fabricate the bare graphite paste sensor (BGPS), 5.0 g of fine graphite powder was mixed with 1.8 mL of paraffin oil to form a homogeneous paste. This paste was then packed into a sensor cavity with an inner diameter of 3.0 mm. The surface was manually polished using clean calque tracing paper until a smooth surface was obtained. The BGPS was immersed in an electrochemical cell containing a suitable supporting electrolyte. After each voltammogram, the sensor surface was renewed by gentle abrasion and re-polishing. For the preparation of the modified graphite paste sensor (MGPS) incorporating 1.0% of the Zn-ZIF-67/0.5 Exf. MMt, 4.95 g of graphite powder was blended with 0.05 g of modifier and 1.8 mL of paraffin oil to produce a uniform paste. The same procedure was followed for the fabrication of the 1.0% (Zn-ZIF-67 framework), (Zn-ZIF-67/0.5Exf. MMt), (Zn-ZIF-67/1.0 Exf. MMt),

### Assessment of point of zero charge (pH_PZC_) of Zn-ZIF-67/0.5 Exf. MMt

To determine the pH point of zero charge (pH_PZC_), a series of NaNO_3_ solutions (0.01 M, 20 mL each) were adjusted to initial pH values ranging from 2.0 to 12.0 using 0.1 M HNO_3_ or NaOH. Each solution was then mixed with 0.06 g of the Zn-ZIF-67/0.5 Exf. MMt nanocomposite and shaken for two days. After filtration, the final pH (pH_f_) of each solution was measured. The pH_pzc_ was determined by plotting the difference between final and initial pH (ΔpH = pH_f_− pH_i_) against the initial pH (pH_i_), as displayed in [Figure S1B].

### Optimal analytical procedures

A SW-AdCSV was performed using both bare and modified sensors in a 10 mL electrochemical cell. The cell contained a 0.5 nM GLY solution in each of BRB, PBS, or 0.1 M HCl as the supporting electrolyte. Measurements were carried out under optimized preconcentration conditions, applying the selected accumulation time followed by a 5-second rest period. Voltammograms were recorded within the potential range of 0.5 to − 0.6 V. To ensure reproducibility and surface renewal, each experiment was repeated five times using fresh electrolyte.

### Collection of real water and soil samples

The Kitchener Drain is the longest agriculture wastewater drain in Egypt, measuring 69 km in total length. It passes through Dakhalia, Gharbia, and Kafrelsheikh, three governorates in the Delta region. The Kitchener Drain runs 46 km through the Kafrelsheikh governorate before draining into the Mediterranean Sea. El-Burullus lagoon and the surrounding area are well-known for aquaculture-related activities in Baltim city (Kafrelsheikh governorate), where fish are farmed utilizing water from the Kitchener Drain [40]. The brackish water sample was obtained from Burullus Lagoon, while the agricultural wastewater sample was sourced from Kitchener Drain (Baltim city, Kafrelsheikh Governorate). Brackish and agricultural wastewater samples were collected in polyethylene containers for analysis using the developed 1.0% (Zn-ZIF-67/0.5 Exf. MMt) MGPS. Water samples were filtered through a 0.45 μm membrane filter to remove insoluble particles. The filtered samples were then diluted with double-distilled water and analyzed promptly after collection to ensure accuracy. Three soil samples were collected at agricultural sites, located near Tanta City, Gharbia Governorate. A 0.5 g portion of each soil sample was spiked with varying concentrations of GLY. This mixture was then placed into a 10 mL micro-electrochemical cell containing 5.0 mL of double-distilled water (DDW) and 5.0 mL of chosen buffer solution. The system underwent sonication for 30 min to facilitate the extraction and homogenization of the analyte. Following sonication, the prepared solution was utilized for subsequent electrochemical analyses.

## Results and discussion

### Characterization of framework and nanocomposites materials

**Fig. 1:**
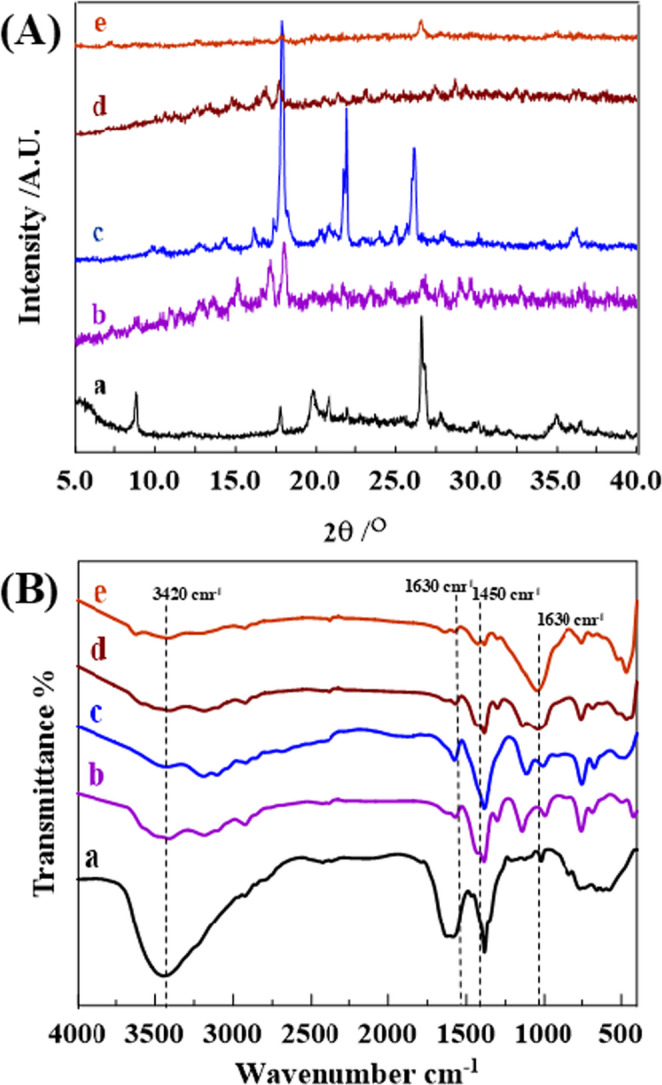
(**A**) XRD patterns, and (**B**) FTIR spectra of (**a**) Na-MMt, (**b**) Zn-ZIF-67 framework, (**c**) Zn-ZIF-67/0.5 Exf. MMt NC, (**d**) Zn-ZIF-67/1.0 Exf. MMt NC, and (**e**) Zn-ZIF-67/2.0 Exf. MMt NC

The XRD patterns **[**Fig. 1A_(a–d)_] provide insight into the structural characteristics of Na-MMt, the pristine Zn-ZIF-67 framework, and its composites with varying Na-MMt content (Zn-ZIF-67/0.5 Exf. MMt, Zn-ZIF-67/1.0 Exf. MMt, and Zn-ZIF-67/2.0 Exf. MMt), respectively. **[**Fig. 1A_(a)_], representing Na-MMt, exhibits basal reflections at approximately 7.2°, 19.8°, and 28.4°, corresponding to the (001), (020), and (110) planes, which confirm its layered silicate structure and crystallinity [48, 49]. **[**Fig. 1A_(b)_], assigned to pristine Zn-ZIF-67, shows diffraction peaks corresponding to 2θ values of approximately 7.3°, 10.4°, 12.9°, 15.3°, 16.8°, 18.0°, 21.8°, 24.9°, 27.0°, 29.0°, 29.9°, 31.4°, and 32.5° attributed to the (011), (002), (112), (022), (013), (222), (114), (233), (224), (134), (044), (334), and (244) planes, respectively. The results obtained from the XRD pattern of the as-synthesized ZIF-67 are consistent with previously reported studies for the experimental [50–52], and simulated XRD patterns of ZIF-67 [50–52], corresponding to the (001), (002), (112), (022), (013), (222), (114), (233), (224), (134), (044), (334), (244) and (235) crystallographic planes. Furthermore, the synthesis of ZIF-67 was confirmed by comparing its characteristic violet color to the white color of ZIF-8 powder [47]. In **[**Fig. 1A_**(c)**_**]**, representing the composite with 0.5 g MMt (Zn-ZIF-67/0.5 Exf. MMt), the ZIF peaks at 18.0°, and 27.0° remain well-defined, suggesting effective dispersion without major disruption to the ZIF framework. In contrast, **[**Fig. 1A_(d & e)_], which corresponds to composites with 1.0 g and 2.0 g MMt (Zn-ZIF-67/1.0 Exf. MMt, and Zn-ZIF-67/2.0 Exf. MMt), display broadening and attenuation of ZIF peaks especially those near 16.8°, and 18.0° indicating reduced crystallinity and possible partial amorphization due to intensified interaction with MMt. MMt reflections, particularly the one near 7.2°, almost disappeared across all composites, confirming the exfoliation of MMt between the formed frameworks. The distinct retention of peak definition in the 0.5 g sample highlights an optimal balance between exfoliation and structural integrity of framework, facilitating effective interfacial interaction while maintaining crystallinity, which may enhance electrochemical or adsorption-related performance.

**Fig. 2:**
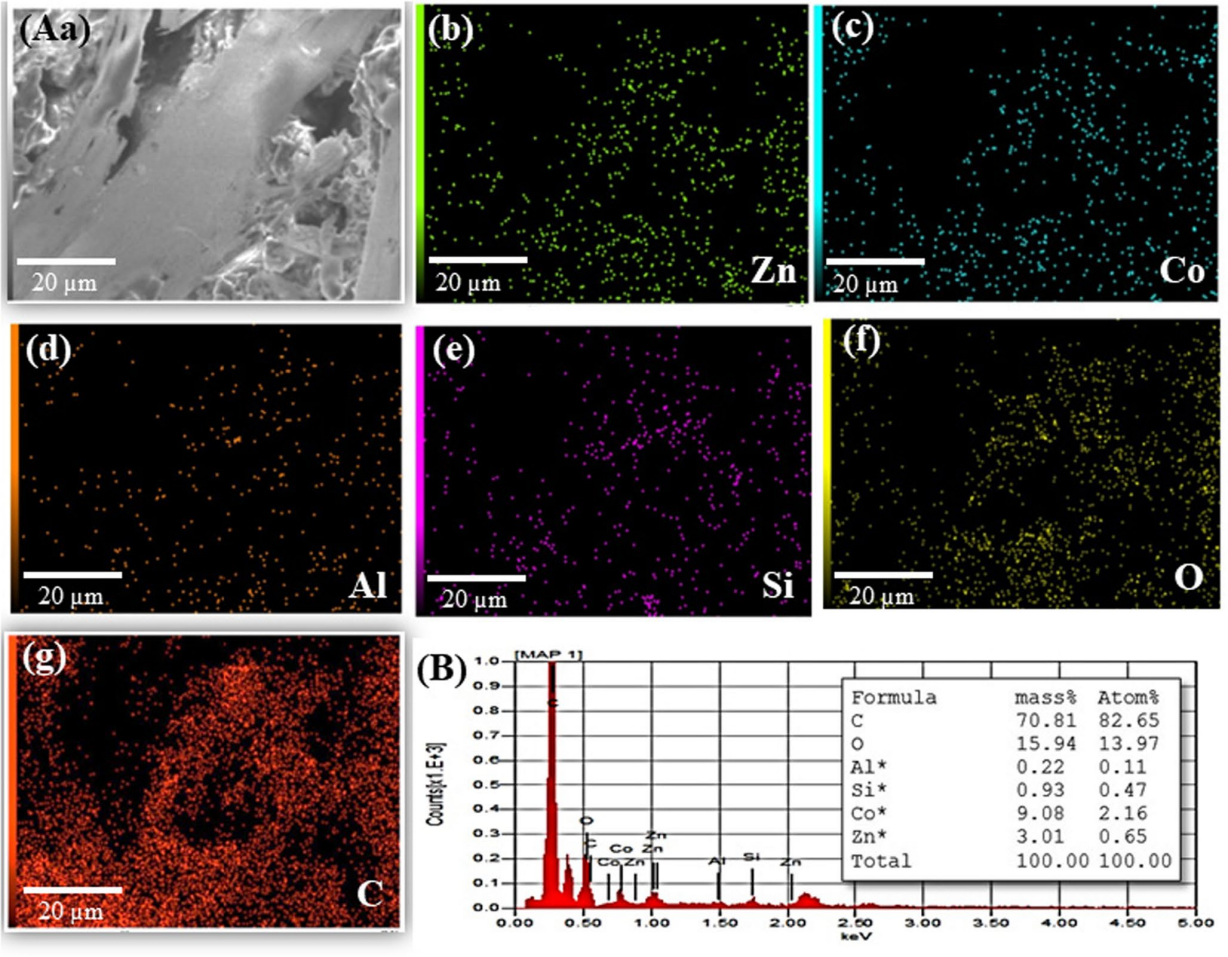
(**A**_(a−g)_) The typical EDX spectrum of the Zn-ZIF-67/0.5 Exf.MMt NC, and (**B**) The EDX elemental mapping distribution of Zn, Co, Si, Al, O, and C elements)

Furthermore, the Fourier transform infrared (FT-IR) spectra of MMt, Zn-ZIF-67, Zn-ZIF-67/0.5 Exf. MMt, Zn-ZIF-67/1.0 Exf. MMt, and Zn-ZIF-67/2.0 Exf. MMt are presented in **[**Fig. 1B**]** for structural analysis. The spectrum of MMt **[**Fig. 1B_a_] displays broad band around 3420 cm⁻¹ corresponding to O–H stretching vibrations, and the peak near 1630 cm^− 1^ is related to H–O–H bending of adsorbed water, which are typical features of MMt [1]. **[**Fig. 2B_(b)_] shows new peaks around 1560–1450 cm^− 1^, which can be assigned to C = N and C = C stretching vibrations of the imidazole ring, along with peaks below 800 cm^− 1^, indicating metal–ligand coordination between Co^2+^/Zn^2+^ and imidazole [2]. In **[**Fig. 1B_(c), (d), and (e)_], representing composites with 0.5, 1.0, and 2.0 g of MMt respectively, the characteristic peaks of both MMt and the Co-Cd imidazole framework are present. The increasing intensity of Si–O stretching vibrations near 1000–1100 cm^− 1^ with higher MMt content confirms successful incorporation, and slight shifts in imidazole-related peaks suggest interactions between the framework and the clay, likely through hydrogen bonding or electrostatic forces [3].

The EDX, and the elemental mapping analyses were performed to examine elemental distribution ratio characteristics of Zn-ZIF-67/0.5 Exf. MMt, as displayed in **[**Figs. 2A_(a−g),_ B]. The SEM-EDX analysis of the Zn-ZIF-67/0.5 Exf. MMt framework showed clear signals for Zn, Co, O and C, confirming the formation of the MOF. A proper peak for Al and Si also appeared, indicating the successful incorporation of MMt into the porous structure of the composite.

**Fig. 3:**
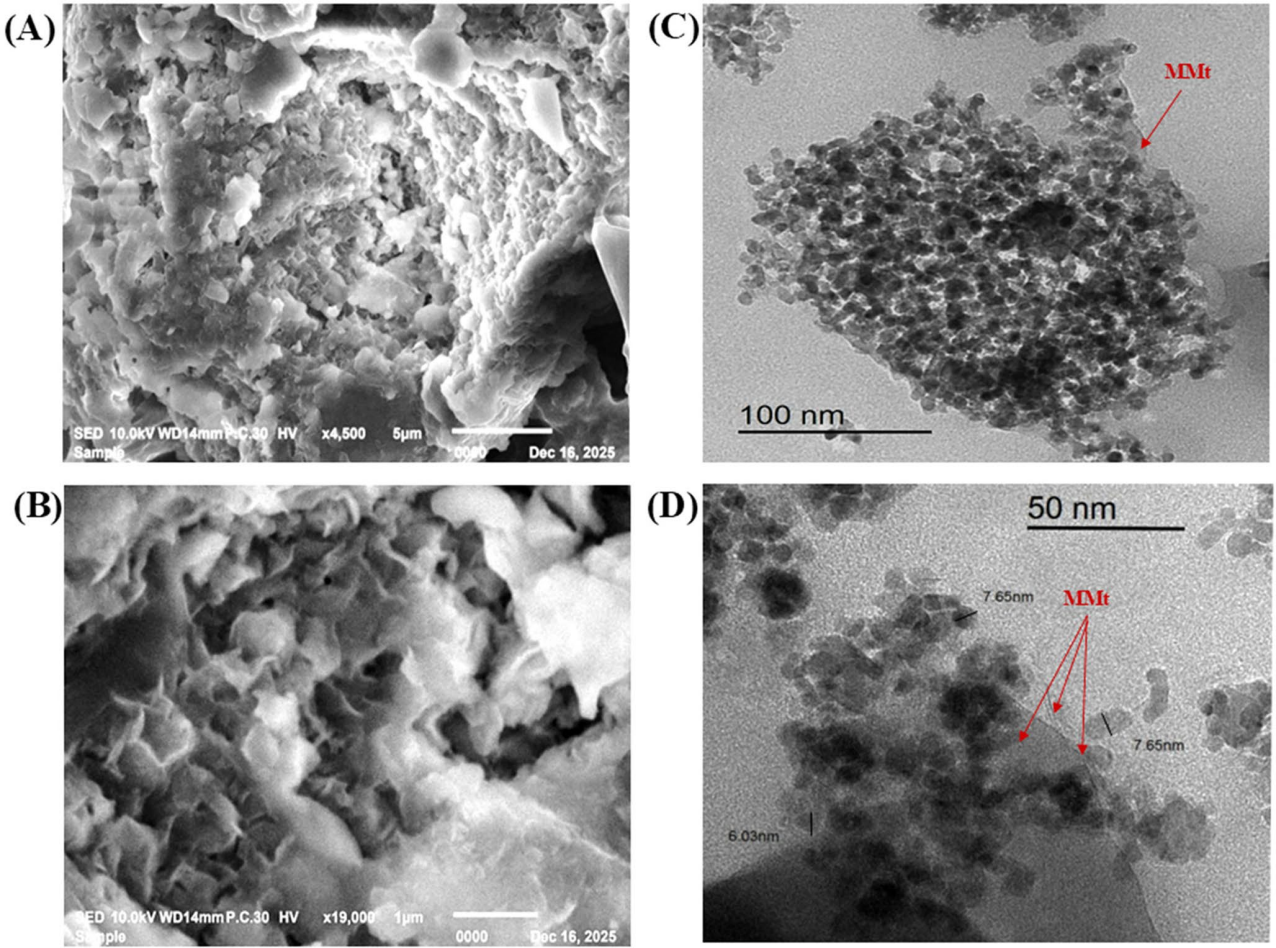
SEM micrographs of Zn-ZIF-67/0.5 Exf. MMt NC at (**A**) low and (**B**) high magnification, and TEM micrographs at (**C**) low and (**D**) high magnification

The SEM micrograph of Zn-ZIF-67/0.5 Exf. MMt **[**Figs. 3A, B**] **reveal a porous and irregular structure formed by aggregated particles of different shapes. The interconnected voids and surface roughness indicate a highly specific surface area, which can enhance mass transport and improve accessibility to active sites. These features reflect a well-developed microstructure that may support superior electrochemical performance, adsorption capacity, or catalytic activity.

Moreover, TEM and BET analyses were performed to examine the morphological characteristics of Zn-ZIF-67/0.5 Exf. MMt, which demonstrated superior crystallinity and structural integrity according to XRD results. These features are essential for enhancing material performance. TEM analysis provided further insight into the morphology of the porous Zn-ZIF-67/0.5 Exf. MMt, as shown in **[**Figs. 3B, C**]**. The TEM images revealed a highly crystalline structure with uniform and dense growth of porous Zn-ZIF-67, even in the presence of exfoliated MMt clay layers. The average particle diameter was approximately 7.11 nm (Image J software). Additionally, [Fig S1A] displays the N_2_ adsorption isotherm of Zn-ZIF-67/0.5 Exf. MMt, which exhibits a typical Type IV curve with a steep increase in adsorption at high relative pressure (P/P⁰), indicating mesoporous characteristics and capillary condensation. The BET surface area (S_BET_) was calculated to be 1450 m²/g, reflecting a highly porous structure with extensive internal surface area.

In addition, the zero point of charge (pH_zpc_) refers to the pH at which the surface charge density of the synthesized material becomes neutral with a net surface charge equals zero. In this study, the pH_zpc_ of Zn-ZIF-67/0.5 Exf. MMt was determined to be 5.5, as shown in [Fig. S1B]. Accordingly, at pH values ≤ 5.5, the surface of Zn-ZIF-67/0.5 Exf. MMt carries a positive charge, while at pH values ≥ 5.5, it becomes negatively charged.

### Investigation of the modified sensor surface in stripping voltammetry

#### Surface electroactivity and resistivity

**Fig. 4:**
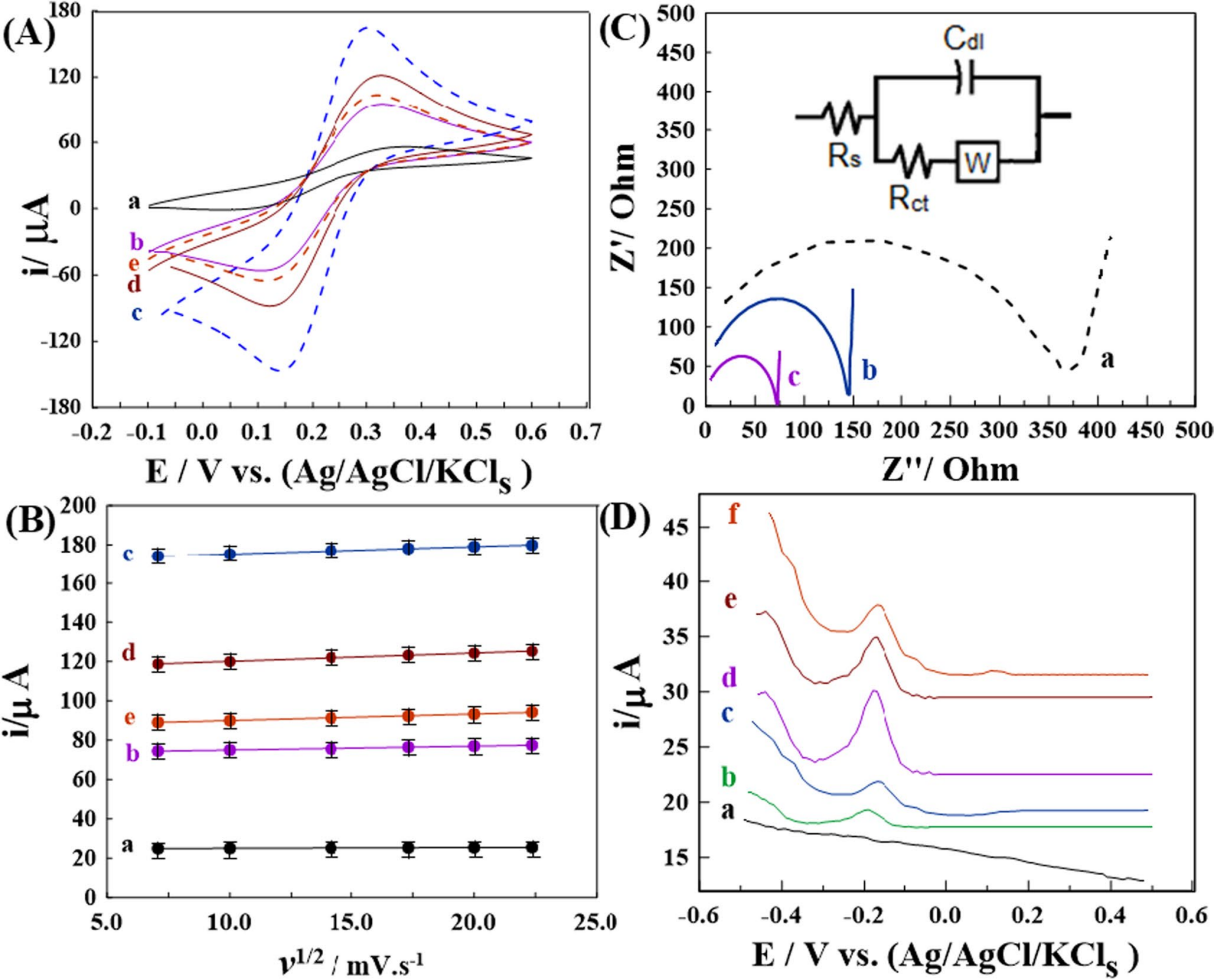
(**A**) CVs of 1.0 mM [Fe(CN)_6_]^3−/4−^ in 0.1 M KCl at 100 mV·s^− 1^, and (**B**) *I*_p_ vs. ν^1/2^ plots from CVs of [Fe(CN)_6_]^3−/4−^ at scan rates of 50–500 mV·s^− 1^ using (**a**) BGPS, (**b**) 1.0% [Zn-ZIF-67], (**c**) 1.0% [Zn-ZIF-67/0.5 Exf. MMt], (**d**) 1.0% [Zn-ZIF-67/1.0 Exf. MMt], and (**e**) 1.0% [Zn-ZIF-67/2.0 Exf. MMt] MGPS.(**C**) Nyquist plots of 1.0 mM K_4_[Fe(CN)_6_] in 0.1 M KCl at 100 mV·s^− 1^ using (**a**) BGPS, (**b**) 1.0% [Zn-ZIF-67] MGPS, and (**c**) 1.0% [Zn-ZIF-67/0.5 Exf. MMt] MGPS.(**D**)Voltammograms for 0.05 nM GLY in 0.1 M HCl (*t*_*acc*_ *= 100 s*,* a = 25 mV*,* f = 80 H*_*z*_, Δ*E*_p_ *= 10 mV*) using: (**a**) BGPS, (**b**) 1.0% Exf. MMt, (**c**) 1.0% [Zn-ZIF-67], (**d**) 1.0% [Zn-ZIF-67/0.5 Exf. MMt], (**e**) 1.0% [Zn-ZIF-67/1.0 Exf. MMt], and (**f**) 1.0% [Zn-ZIF-67/2.0 Exf. MMt] MGPS

To gain deeper insight into the sensing behavior of the modified stripping voltammetric sensors, the electroactive surface area of each synthesized sensor was assessed. The CV was performed using a 1.0 mM solution of (K_4_[Fe(CN)_6_]) in 0.1 M KCl as the redox probe, with a scan rate (*v*) of 100 mV·s^− 1^. **[**Figure 4A**]** presents the CV profiles for Bare GPS (BGPS), 1.0% [Zn-ZIF-67] MGPS, 1.0% [Zn-ZIF-67/0.5 Exf. MMt] MGPS, 1.0% [Zn-ZIF-67/1.0 Exf. MMt] MGPS, and 1.0% [Zn-ZIF-67/2.0 Exf. MMt] MGPS. All proposed sensors exhibited distinct redox peaks, confirming a quasi-reversible electron transfer for the [Fe(CN)₆]^3−^/^4−^ couple. The peak-to-peak separation (Δ*E*_p_) values were notably reduced in the modified sensors 190, 150, 190, and 210 mV for 1.0% [Zn-ZIF-67], 1.0% [Zn-ZIF-67/0.5 Exf. MMt], 1.0% [Zn-ZIF-67/1.0 Exf. MMt], and 1.0% [Zn-ZIF-67/2.0 Exf. MMt] MGPSs compared to 240 mV for BGPS. This reduction indicates improved electron transfer kinetics, likely due to enhanced charge density and conductivity at the sensor surfaces. Additionally, the voltammogram of 1.0% [Zn-ZIF-67/0.5 Exf. MMt] MGPS showed the highest peak current among the tested sensors, reflecting superior sensitivity. The enhanced redox response can be attributed to the synergistic contribution of Zn^2+^ and Co^2+^ ions, which provide an optimal balance of surface area and electrical conductivity [53]. In addition, the interaction between Zn-ZIF-67 and exfoliated montmorillonite increases surface roughness and facilitates electron mobility, further improving sensor performance. However, at higher Exf. MMt loadings, a slight decrease in current is observed, likely due to reduced conductivity or partial aggregation, which can limit the efficiency of the sensor [44].

To quantify the electroactive surface area (*A*_s_), additional cyclic voltammetry (CV) measurements were carried out using a 1.0 mM of K_4_[Fe(CN_6_)] in 0.1 M of KCl over a scan rate range (*v*) of 50–500 mV·s^− 1^. The anodic peak current (*I*_p_) was plotted against the square root of the scan rate (ν^1/2^), as illustrated in **[**Fig. 4B**]**. Based on the Randles–Sevcik equation [54], the calculated *A*_s_values were 0.054, 0.27, 0.68, 0.57, and 0.45 cm^2^ for the BGPS 1.0% [Zn-ZIF-67], 1.0% [Zn-ZIF-67/0.5 Exf. MMt], 1.0% [Zn-ZIF-67/1.0 Exf. MMt], and 1.0% [Zn-ZIF-67/2.0 Exf. MMt] MGPSs, respectively. These results indicate a substantial improvement in electroactive surface area, with the 1.0% [Zn-ZIF-67/1.0 Exf. MMt] MGPS exhibiting a 12.5-fold increase compared to the unmodified sensor.

Moreover, EIS was employed to evaluate the charge transfer resistance (*R*_ct_) at the sensor electrolyte interface. Nyquist plots were recorded for 1.0 mM [Fe(CN)_6_]^3−/4−^ in 0.1 M KCl at 100 mV·s^− 1^ over a frequency range of 0.1 H_z_ to 10,000 H_z_, as shown in **[**Fig. 4C**]**. The *R*_ct_ values extracted from the plots were 370 Ω for BGPS, 145 Ω for 1.0% [Zn-ZIF-67] MGPS, and 75.0 Ω for 1.0% [Zn-ZIF-67/0.5 Exf. MMt] MGPS. Notably, the 1.0% [Zn-ZIF-67/0.5 Exf. MMt] MGPS demonstrated the lowest *R*_ct_ value, signifying enhanced electron transfer kinetics and reduced interfacial impedance. These findings corroborate their superior electrochemical performance and improved sensing efficiency.

Furthermore, as illustrated in **[**Fig. 4C; (inset)], the equivalent circuit modeling of (R(C(R_W_))) was employed to correlate the EIS for 1.0% [Zn-ZIF-67/0.5 Exf. MMt] MGPS. This model comprises series components, including the bulk solution resistance, R_s_, and a parallel combination of the double layer capacitance, C_dl_, charge transfer resistance, R_ct_, and Warburg impedance, W.

The high-frequency intercept, as depicted in the equivalent circuit inset, is determined by *R*s, which encompasses the electrolyte resistance, the intrinsic resistance of the electrode surface, and the contact resistance. The diverse high-frequency intercepts observed in the Nyquist plots can be predominantly attributed to fluctuations in solution and interfacial resistances and/or alterations in surface roughness and heterogeneity due to electrode modification (i.e., modified electrochemical sensors often do not originate from the same points in Nyquist plots because electrode modification affects interfacial resistance and capacitance, not solely the charge-transfer process). The high-frequency intercept is therefore no longer identical, even in the same electrolyte.

#### Preliminary electrochemical evaluation of the prepared sensors

The SW-AdCSV technique was used to detect 0.05 nM GLY in 0.1 M HCl, as shown in **[**Fig. 4D**]**. The accumulation potential (*E*_acc_) and time (*t*_acc_) were set at 0.5 V and 100 s, respectively. **[**Figure 4D**]** shows that MGPSs modified with 1.0% Exf. MMt,1.0% Zn-ZIF-67, and with increasing amounts of Exf. MMt specifically 0.5, 1.0, and 2.0 g exhibited a reduction peak at −0.18 V, corresponding to the electrochemical reduction of GLY to methylamine and inorganic phosphate [55]. Noteworthy, the highest affinity towards GLY and well-defined reduction peak current was acquired utilizing 1.0% [Zn-ZIF-67/0.5 Exf. MMt] MGPS with about three-fold increase with respect to the 1.0% Exf. MMt, and 1.0% Zn-ZIF-67 **[**Fig. 4D**]**. This enhancement is attributed to the porous structure of Zn-ZIF-67, which facilitates electron transfer, and to the adsorption capacity of the neutral MMt structure [56, 57]. Additionally, improved distribution of active sites and increased electroactive surface area contribute to the stronger signal [58]. This is confirmed by the absence of the reduction peak of GLY at the unmodified BGPS **[**Fig. 4D, curve a]. Furthermore, The strong adsorption of 1.0% [Zn-ZIF-67/0.5 Exf. MMt] MGPS toward GLY was verified by cyclic voltammetry using 0.1 nM GLY in 0.1 M HCl, as displayed in [Figure S2A, B]. A linear relationship between log ip and log ν was obtained, with a slope of 0.819 µA·mV⁻¹·s (*R* = 0.995). This value is very close to the theoretical slope of 1.0, confirming that the process is mainly adsorption-controlled with some diffusion contribution [59].

#### Mechanism of the reaction upon the surface of sensor

**Scheme 3:**
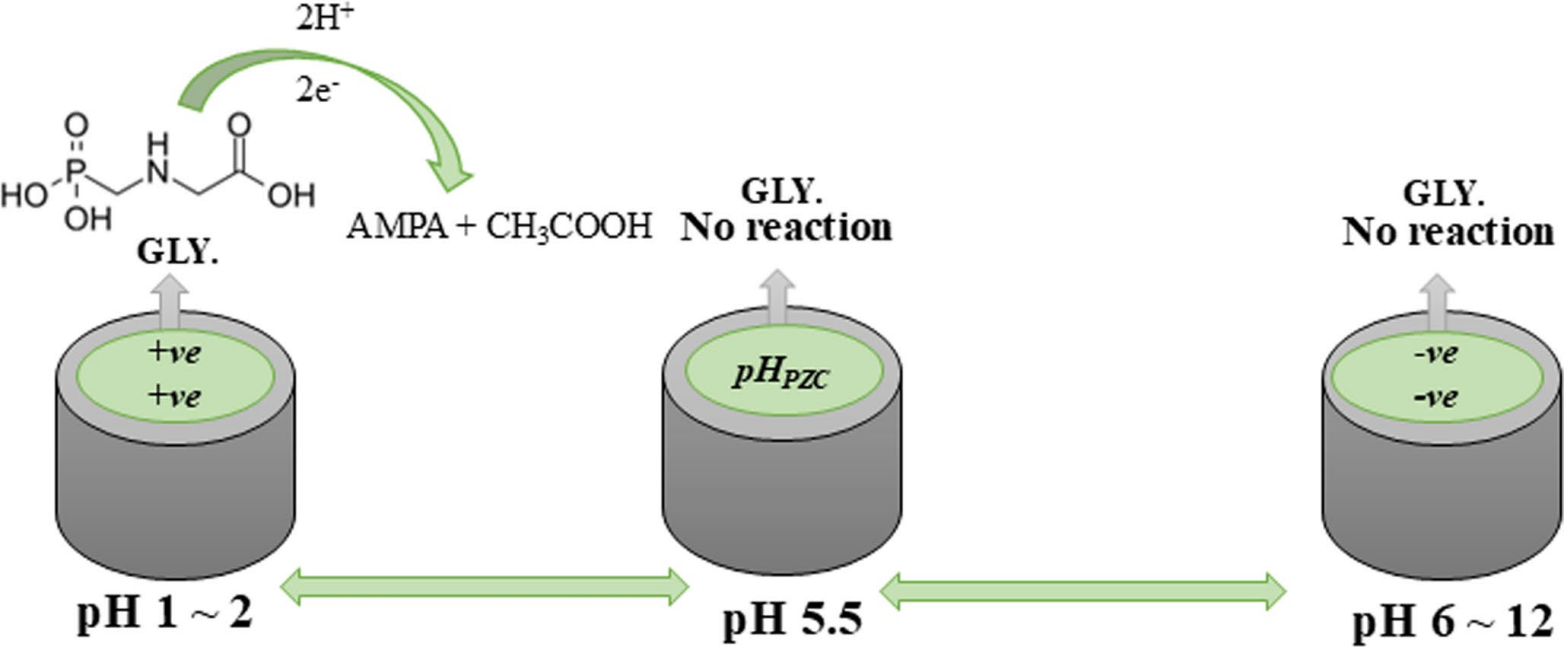
Proposed mechanism for GLY reduction at 1.0% [Zn-ZIF-67/0.5 Exf. MMt] MGPS

The electrochemical reduction of GLY on the 1.0% [Zn-ZIF-67/0.5 Exf. MMt] MGPS proceeds through multiple steps, as displayed in **[**Scheme 3**]**. Initially, electron transfer at the cathode breaks the carbon–nitrogen (C–N) bond, producing aminomethylphosphonic acid (AMPA) [55]. The Zn-ZIF-67 component offers a high surface area and porosity, which enhances adsorption and electron transfer, while the exfoliated MMt improves charge stabilization and dispersion [44, 60].

The electrochemical behavior of the Zn-ZIF-67/0.5 Exf. MMt composite is strongly affected by its surface charge, which is determined by its pH_PZC_, approximately at pH 5.5, as mentioned before. Below this pH, the MMt component becomes (+ *ve*) charged due to protonation of aluminum hydroxide species (Al(OH)_3_ + H^+^ → AlOH_2_^+^), enhancing its attraction to (-*ve*) charged analytes [44]. At pH values ≥ 5.5, hydroxide ions neutralize the surface by converting AlOH_2_^+^ to Al(OH)_2_ and simultaneously deprotonate the Zn-ZIF-67 framework. This deprotonation forms ligand-OH^−^ species coordinated to Zn centers and releases electrons, contributing to a (+ *ve*) charge on the MOF. These surface changes directly influence the interaction of GLY with the surface of sensor, which contains four ionizable groups (pK_a_~ 0.8, ~ 2.3, ~ 6.0, and ~ 11.0) [61]. At pH ˂ 2 (0.1 M HCl), the ionizable groups of GLY with pK_a_ values ˃ 2 are mostly protonated, resulting in a neutral or slightly negative overall charge (phosphonic and carboxylic acid groups) [62]. This charge profile enhances electrostatic interaction with the (+ *ve*) MMt surface, which supports stronger adsorption and improves electron transfer, as displayed in **[**Scheme 3**]**.

### Optimizing square wave voltammetry and adsorptive stripping parameters

#### Influence of pH and buffer composition on electrochemical performance

The efficiency of GLY detection using cathodic stripping voltammetry is strongly affected by the type and concentration of the supporting electrolyte, which influence the electrochemical behavior at the sensor surface. To determine optimal medium, voltammetric responses of 0.05 nM GLY were measured across a range of pH values using BRB upon the surface of a 1.0% [Zn-ZIF-67/0.5 Exf. MMt] MGPS. Among the tested supporting electrolytes, only pH 2 produced a distinct and well-defined peak current. No measurable response was observed at increased pH values, indicating that strongly acidic environments are critical for effective GLY detection.

Moreover, the SW-AdCSV detection of 0.05 nM GLY in 0.1 M HCl (pH 1) offers superior sensitivity compared to BRB and PBS (pH 2) [63], as displayed in [Figure S3A]. This is attributed to the high proton concentration in HCl, which enhances electron transfer kinetics and promotes the reduction of the functional groups of GLY, leading to stronger and more distinct voltammetric signals [12]. In contrast, buffered systems like BRB and PBS may introduce electroactive species that interfere with the sensor response and reduce sensitivity through surface adsorption. Additionally, acidic media such as HCl minimize background current and improve the stability of 1.0% [Zn-ZIF-67/0.5 Exf. MMt] MGPS, which are crucial for ultra-trace detection. These benefits align with the principles of stripping voltammetry, which is widely used for trace analysis due to its high sensitivity and low detection limits.

The pronounced electrochemical peak observed in HCl is primarily due to favorable surface interactions and efficient electron transfer. At pH values below the point of zero charge (pH_PZC_ ≈ 5.5), the sensor surface becomes positively charged due to protonation of Al(OH)_3_ sites in MMt to AlOH₂⁺ [44], and protonation of imidazole groups in Zn-ZIF-67 [64]. Under these acidic conditions, GLY remains partially deprotonated, retaining negatively charged phosphonic and carboxylic acid groups. This allows for strong electrostatic attraction between GLY and the positively charged sensor surface [62]. Moreover, Zn^2+^ ions within the ZIF framework can coordinate with the functional groups of the GLY, enhancing adsorption [65]. The porous architecture of Zn-ZIF-67 facilitates rapid diffusion and effective electron transfer, while the acidic medium stabilizes reactive species and minimizes interference, resulting in a sharp and well-defined voltammetric signal. In contrast, at pH values above 2, GLY undergoes further deprotonation, acquiring additional negative charges. Simultaneously, the sensor surface becomes less positively charged or even negatively charged near or above its pH_PZC_. This leads to electrostatic repulsion between GLY and the surface of sensor, hindering analyte accumulation and suppressing the voltammetric response. Furthermore, buffers commonly used at higher pH, such as phosphate and universal buffers, may chelate Zn^2+^ ions or contribute to background currents, further compromising sensor performance.

#### Effect of square wave voltammetry parameters and accumulation conditions on electrochemical performance

**Fig. 5:**
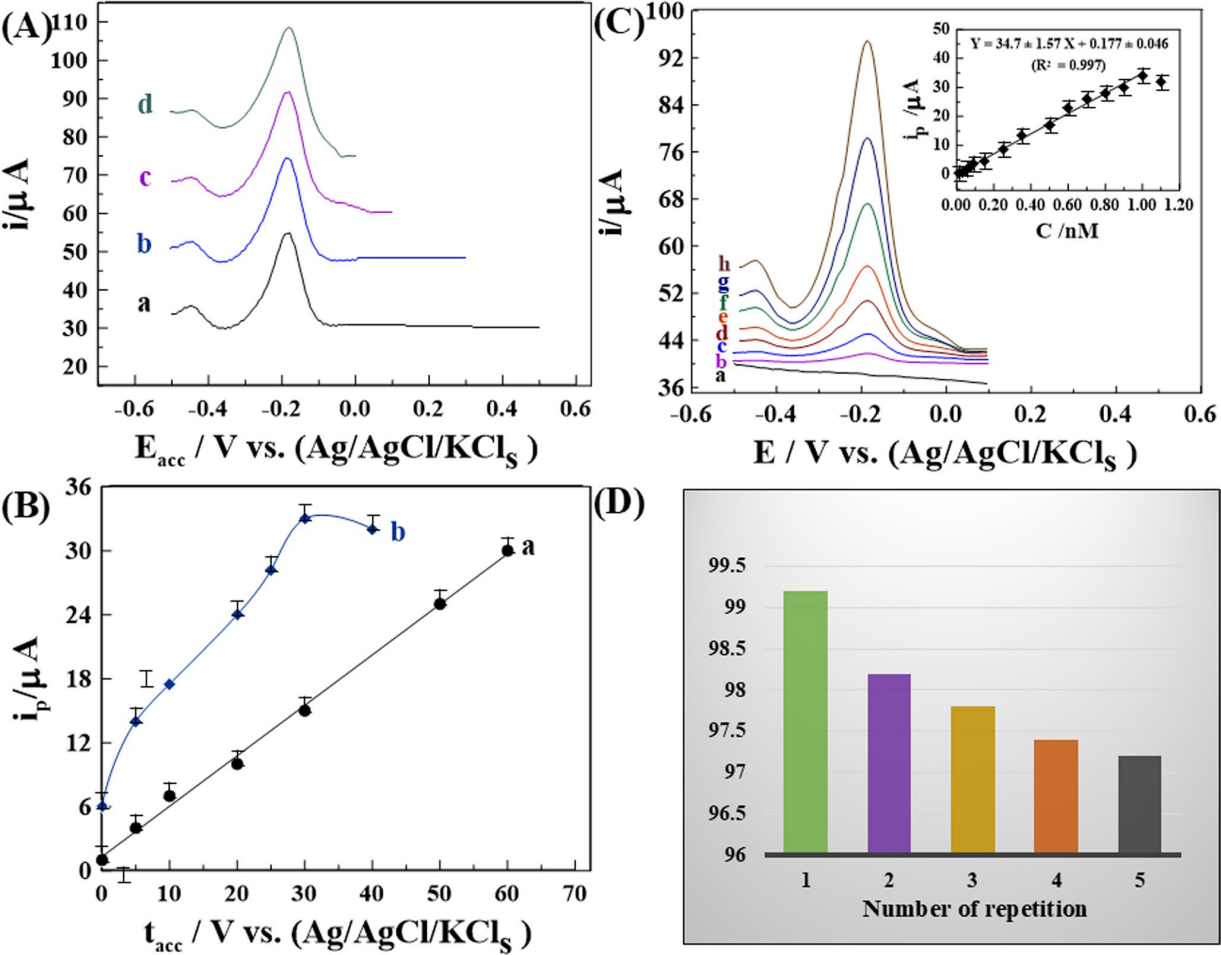
(**A**) Effect of changing of *E*_*acc*_ of 0.05 nMGLY, and (**B**) The effect of changing ***t***_***acc***_ of 0.09 and 0.8 nM of GLY in 0.1 M HCl at 1.0% [Zn-ZIF-67/0.5 Exf. MMt] MGPS (ΔE_***s***_= 7 mV, *f* = 120 H_z_, and ***a*** = 30 mV). (**C**) SW–AdCV voltammograms of different amounts of GLY in in 0.1 M HCl on 1.0% [Zn-ZIF-67/0.5 Exf. MMt] MGPS (***E***_***acc***_= 0.1 V, ***t***_acc_= 25 s, ***Δ***E_***s***_= 7 mV, ***f*** = 120 H_z_, and ***a*** = 30 mV) in bulk form: (**a**) baseline, (**b**) 0.05, (**c**) 0.09, (**d**) 0.15, (**e**) 0.25, (**f**) 0.35, (**g**) 0.5, and (**h**) 0.8 nM (inset: its corresponding plot (*n* = 3)). (**D**) Histogram of intra-day precision of 0.09 nM GLY in 0.1 M HCl on the surface of 1.0% [Zn-ZIF-67/0.5 Exf. MMt] MGPS

As shown in [Figure S3B–D], the optimal pulse parameters were determined to be frequency ***(f)*** = 120 Hz, pulse amplitude ***(a)*** = 30 mV, and scan increment ***(∆E***_***s***_***)*** = 7 mV for the 1.0% [Zn-ZIF-67/0.5 Exf. MMt] MGPS. The highest peak current intensity was observed at an accumulation potential (***E***_acc_) of 0.1 V **[**Fig. 5A**]**. Additionally, the effect of accumulation time (***t***_**acc**_) on the peak current for 0.09 and 0.8 nM GLY was evaluated using the above parameters on the same modified sensor **[**Fig. 5B**]**. The peak current response increased linearly with *t*_acc_ up to 60 s for 0.09 nM and 30 s for 0.8 nM, followed by a significant decline, likely due to surface saturation by GLY species. Based on these findings, the optimal accumulation conditions for GLY detection were established as *E*_acc_= 0.1 V and *t*_acc_= 25 s, which were applied in subsequent analytical experiments.

### Method validation

#### Linearity range (LR) and limit of detection (LOD)

As depicted in **[**Fig. 5C**]**, SW-AdCSV calibration plot of *I*_p_ vs. different concentrations of GLY were recorded under the foregoing standard parameters upon the 1.0% [Zn-ZIF-67/0.5 Exf. MMt] MGPS. The *I*_p_ of the peak linearly increased in the range of 0.01 −1.0nM of GLY with R^2^ = 0.996. Furthermore, the constructed sensor achieved nano-level of limit of detection (LOD = 0.003 nM) with sensitivity (34.7 µA.nM^− 1^) and covering broader linearity range of (LR = 0.01 −1.0 nM) in compared to the recent reported electro-analytical methods [25–34, 66], which used for detection of GLY in various water, food, drinks and soil samples, as mentioned in **[**Table 1**]**.

Specially, our sensor achieves highly sensitive GLY detection in soil with a limit of detection (LOD) of 0.009 nM and a linearity range of 0.03–1.0 nM, outperforming other reported methods such as silane-smectite (LOD: 0.98 µM, range: 10–100 µM) [26], MWCNT/CuNP/Py (LOD: 0.002 µM, range: 0.01–1.0 µM) [25], and SERS-based substrates (LOD: 3 µg/L) [30]. This enhanced sensitivity, combined with direct detection in untreated soil samples, ensures more accurate monitoring of GLY contamination at ultra-trace levels. Moreover, in comparison to previously reported sensors applied to aqueous and agricultural matrices [28, 29, 31–34, 66], our sensor exhibits markedly enhanced analytical performance, achieving an ultra-low limit of detection (0.009 nM) and a narrow linear range (0.03–1.0 nM). Unlike other methods that are limited to water, juice, or crop extracts and show higher detection limits such as 0.52 µg/mL (3075 nM) [27], 0.13 ppm (769 nM) [28], 2 µg/L (11.83 nM) [29], 2.84 µM (2840 nM) [66], 3 µg/L (17.74 nM) [30], 1 µmol/L (1000 nM) [31], 3.53 µM (3530 nM) [32], 2 µM (2000 nM) [33], 0.96 µM (960 nM) [34], 0.8 pg/mL (4.73 nM) [67], 2.7 µmol/L (2700 nM) [68], and 1.2 ng/mL (7.10 nM) [69], our sensor enables direct analysis of untreated soil samples, which are inherently more complex and less represented in GLY monitoring. While SERS [30] and LSPR [32] platforms offer high sensitivity and reusability, they require intricate fabrication and are less compatible with solid-phase detection. Similarly, Spectro-electrochemical, graphene-based, and ZnO-based sensors [31, 33, 34, 68, 69] provide rapid detection in water but lack the matrix adaptability and ultra-trace capability demonstrated in our study. These comparative advantages highlight the practical utility of our sensor for environmental applications requiring high sensitivity, minimal sample preparation, and robust performance in heterogeneous media.

#### Assessment of the sensor reliability, repeatability, and stability

The reliability and repeatability of sensors were evaluated under optimized conditions using intra-day and inter-day analyses. Intra-day precision was assessed by recording SW-AdCS voltammograms of 0.09 nM GLY in 0.1 M HCl with five freshly prepared 1.0% [Zn-ZIF-67/0.5 Exf. MMt] MGPSs, measured in parallel on the same day. Inter-day performance was examined over three consecutive days. The mean recovery and relative standard deviation (R% ± RSD) were 97.87% ± 0.76 for intra-day precision [Figures S4A,5D] and 97.8% ± 2.19 for inter-day analysis [Figures S4B,6 A], confirming high reliability and reproducibility. Long-term stability was further investigated by storing the modified electrodes at ambient conditions and testing them every 7 days over a 30-day period (*n* = 3). The sensor retained 97.30% of its initial response after 7 days, 96.70% after 15 days and 93.40% after 30 days, indicating strong signal retention [Figures S4C, 6B]. As summarized in **[**Table 2**]**, these findings demonstrate the robust reliability of sensor, consistent repeatability, and excellent storage stability, largely attributed to the chemical stability of the 1.0% [Zn-ZIF-67/0.5 Exf. MMt] MGPS.

**Table 2:** The intra and inter-day assay of 0.09 nM GLY in its pure form utilizing the SW-AdCSV method:

	C_Taken_(nM)			C_Found_(nM) ± SD	Recovery ± Precision (*R* % ± RSD %)		Relative Error E_*r*_ (%)
Intra-day analysis		0.09	0.0880 ± 0.005		97.87 ± 0.76	−2.08	
Inter-day analysis		0.09	0.0881 ± 0.008		97.80 ± 2.19	−2.20	
Stability _(*n* = 3)_*							
7 days 15 days 30 days		0.09	0.089 ± 0.003 0.087 ± 0.008 0.089 ± 0.020		97.30 ± 1.16 96.70 ± 2.34 93.40 ± 3.60	−2.70 −3.30 −6.60	

* (n = 3) means three measurements.

Moreover, the 1.0% [Zn-ZIF-67/0.5 Exf. MMt] MGPS was applied for the determination of 0.5 nM GLY in 0.1 M HCl for five replicates (*n* = 5) and [11] a mean R% ± RSD of 99.31% ± 1.06 was achieved. The obtained results were compared with those obtained from the reported chromatographic method [11], which involved pre-column derivatization with the fluorescent reagent 9-fluorenylmethylcloroformate (FMOC), followed by large-volume injection in a coupled-column LC system using fluorescence detection (LC–LC–FD). This method achieved R% ± RSD of 98.88% ± 1.31. The developed 1.0% [Zn-ZIF-67/0.5 Exf. MMt] MGPS and the described LC method [11] were statistically compared using both *F-statistics* and the *t-test* at the 95% confidence level. The calculated *F-statistics* of 1.51 does not exceed the theoretical value of (6.39), indicating no significant difference with respect to precision. Additionally, the computed *t-test* of 0.58 does not surpass the expected value of (2.31), suggesting that there is no discernible variation in accuracy.

### Selectivity

**Fig. 6:**
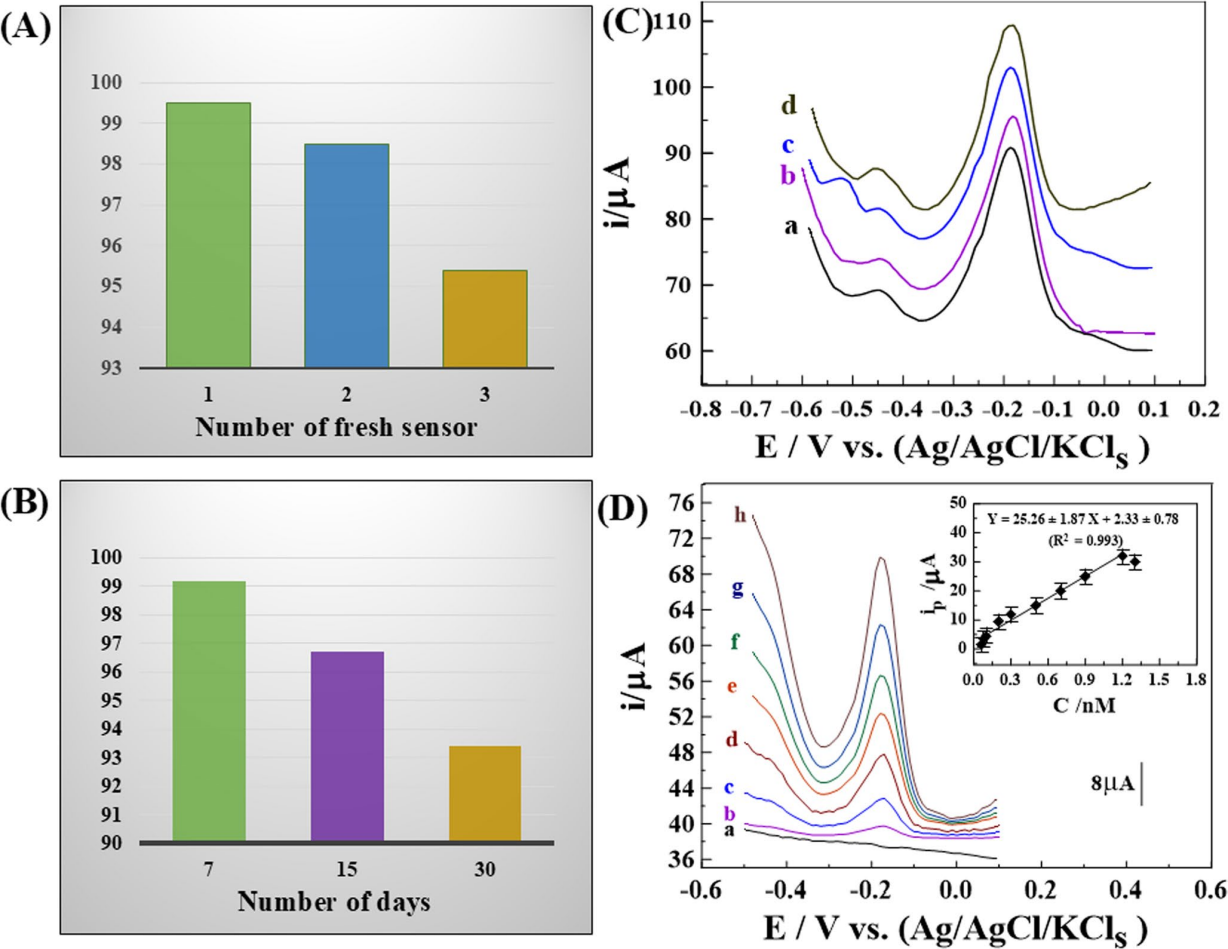
Histogram of (**A**) repeatability, and (**B**) stability of 0.09 nM GLY in 0.1 M HCl on the surface of 1.0% [Zn-ZIF-67/0.5 Exf. MMt] MGPS. (**C**) SW–AdCV voltammograms of (**a**) 0.05 nM GLY, (**b**) 0.05 nM GLY + 5.0 nM Mix_1_, (**c**) 0.05 nM GLY + 5.0 nM NiCl_2_, and (**d**) 0.05 nM GLY + 0.05 nM AMPA in 0.1 M HCl on 1.0% [Zn-ZIF-67/0.5 Exf. MMt] MGPS (***E***_***acc***_= 0.1 V, ***t***_acc_= 25 s, ΔE_***s***_= 7 mV, ***f*** = 120 H_z_, and ***a*** = 30 mV) (*n* = 3). (**D**) SW–AdCV voltammograms of different s of GLY in 0.1 M HCl on 1.0% [Zn-ZIF-67/0.5 Exf. MMt] MGPS (***E***_***acc***_= 0.1 V, ***t***_acc_= 25 s, ΔE_***s***_= 7 mV, ***f*** = 120 H_z_, and ***a*** = 30 mV) in Soil_1_ sample: (**a**) baseline, (**b**) 0.06, (**c**) 0.1, (**d**) 0.2, (**e**) 0.5, (**f**) 0.7, (**g**) 0.9, and (**h**) 1.2 nM (inset: its corresponding plot (*n* = 3))

The anti-interference performance (selectivity) of 1.0% [Zn-ZIF-67/0.5 Exf. MMt] MGPS was investigated by the addition of common interfering species in water and soil samples, as displayed in **[**Fig. 6C**]**. Whereas the ***i***_*p*_ voltammogram of 0.05 nM GLY was evaluated after addition of 5.0 nM (~ 100-fold) of major cations and anions such as (Mix_1_: K^+^, Na^+^, Ca^+ 2^, Mg^+ 2^, Mg^2+^, Fe^3+^, Al^3+^, Zn^2+^, Ni^2+^, Cr^3+^, NH_4_^+^, Cl^−^, SO_4_^2−^, NO_3_^−^ and HCO_3_^−^) at 0.1 M HCl supporting electrolyte. Notably, the ions including Ca²⁺, Mg²⁺, Al³⁺, Zn²⁺, Cr³⁺, Na⁺, K⁺, Cl⁻, SO₄²⁻, NO₃⁻, and HCO₃⁻are considered non-electroactive in this range, contributing primarily to ionic strength or background conductivity without producing detectable peaks, as illustrated in **[**Fig. 6C_b_]. While Ni²⁺ may show a weak signal near − 0.52 V, though often suppressed under acidic conditions [70], as displayed in **[**Fig. 6C_c_]. Conversely, the GLY primary natural decomposition pathway occurs through degradation by soil microfora under both aerobic and anaerobic conditions [71]. The main deactivation path is hydrolysis to AMPA. [Fig. 6C_d_] represents the SW voltammogram of 0.05 nM of each of GLY and AMPA.No additional peaks corresponding to the GLY metabolite AMPA were observed when SW-AdCSV was applied at the 1.0% [Zn-ZIF-67/0.5 Exf. MMt] MGPS under the optimized conditions, confirming the good selectivity of the developed sensor for GLY quantification in real soil samples. This behavior can be attributed to the fact that AMPA, unlike many small organic molecules, exhibits very low intrinsic cathodic activity under conventional voltammetric conditions. Owing to the strong C–P bond and its high degree of protonation, analytical electrochemical studies generally do not report a distinct reduction peak for AMPA prior to the onset of the hydrogen evolution reaction in acidic media when using an Ag/AgCl reference electrode. Consequently, electrochemical determination of AMPA is typically based on anodic or complexation-assisted signals rather than cathodic reduction peaks, underscoring the absence of a well-defined cathodic response under typical voltammetric conditions [72, 73].Furthermore, **[**Fig. 6C**]** demonstrated that there is no substantial variation in the peak current of 0.05 nM GLY, so affirming the strong selectivity of 1.0% [Zn-ZIF-67/0.5 Exf. MMt] for GLY analysis in real samples without interferences from the matrix.

### Application in environmental samples

Under optimized analytical conditions, SW-AdCS voltammograms and the corresponding calibration curve were recorded for GLY detection in 0.05 nM GLY in 0.1 M HCl using a 1.0% [Zn-ZIF-67/0.5 Exf. MMt] MGPS surface. A spiked soil sample (Soil_1_) was added directly without pre-treatment. As shown in **[**Fig. 6D**]**, the sensor exhibited an LR from 0.05 to 1.2 nM, with a sensitivity of 25.26 µA. nM^− 1^. The calibration curve followed the equation: i_p/µA_ = 25.26 ± 1.87 C_GLY/nM_ + 2.33 ± 0.78 with a high correlation coefficient (R^2^ = 0.993) and a limit of detection (LOD) of approximately 0.015nM. All investigated real samples (brackish water, agricultural wastewater and two soil samples) were analyzed applying the developed sensor and no GLYwere detected. Therefore, the collected real samples were spiked with two concentration levels of 0.09 nM and 0.5 nM (0.03746 ppb) GLY and the SW-AdCS voltammograms were recorded in these matrices. As summarized in **[**Table 3**]**, the sensor achieved satisfactory recovery (R%) and low relative standard deviation (RSD%), indicating minimal matrix interference. These results confirm the accuracy and reliability of the sensor for GLY detection in complex environmental samples.

**Table 3:** Detection of GLY carried out in different real samples (*n* = 3)

Sample	C_Added_(nM)		C_Found_(nM) ± SD		*R* % ± RSD%	RE%
Brackish Water	0.09	0.0895 ± 0.004		99.44 ± 1.87		−0.62
	0.5	0.4958 ± 0.087		99.16 ± 2.20		−0.86
Agricultural Wastewater	0.09	0.0892 ± 0.004		99.12 ± 1.34		−0.88
	0.5	0.4943 ± 0.008		98.86 ± 2.15		−1.14
Soil _2_	0.09	0.0884 ± 0.008		98.22 ± 2.12		−1.78
	0.5	0.4888 ± 0.012		97.76 ± 3.57		−2.25
Soil _3_	0.09 0.5	0.0908 ± 0.006 0.5030 ± 0.011		100.9 ± 3.24 100.6 ± 2.30		0.90 0.60

## Conclusion

A porous Zn-ZIF-67 was synthesized and integrated with varying amounts of exfoliated Exf. MMt via a solvothermal method. The resulting nanocomposite exhibited a high specific surface area (1450.0 m²/g) and a crystalline size of 7.11 nm, as confirmed by TEM analysis. The synthesized nanocomposite was used as an electrode modifier to develop a voltammetric sensor for GLY. The optimized modified graphite paste sensor demonstrated enhanced electroactive surface area, faster electron transfer kinetics, and improved adsorption capacity compared to the unmodified sensor. This sensor enabled highly sensitive and reliable detection of GLY in brackish water, agricultural wastewater, and soil samples using SW-AdCSV. Its strong electrochemical performance allows for direct analysis of herbicides and pesticides in environmental samples without the need for complex pretreatment steps. The developed 1.0% [Zn-ZIF-67/0.5 Exf. MMt] MGPS demonstrates clear novelty compared with previously reported electrochemical sensors by offering a balanced and simultaneous improvement in terms of sensitivity, selectivity, stability, cost, and ease of fabrication.

## Supplementary Information

Below is the link to the electronic supplementary material.

Supplementary File 1 (DOCX 344 KB)

## Data Availability

All data used or produced in this study are presented within this work.

## References

[1] Öztekin R, Sponza DT, The effect of dissolved natural organic matter (NOM) on the photocatalytic removal of 4-Chloro-2-Methylphenoxyacetic acid (MCPA) endocrine disrupting compound from the surface water using carbon Nanotubes/Titanium dioxide (CNT-TiO_2_)

[2] Benbrook CM (2016) Trends in glyphosate herbicide use in the United States and globally. Environ Sci Eur 28:3 27752438 10.1186/s12302-016-0070-0 PMC5044953

[3] Singh S, Kumar V, Gill JPK, Datta S, Singh S, Dhaka V, Kapoor D, Wani AB, Dhanjal DS, Kumar M (2020) Herbicide glyphosate: toxicity and microbial degradation. Int J Environ Res Public Health 17:7519 33076575 10.3390/ijerph17207519 PMC7602795

[4] Sok V, Fragoso A (2019) Amperometric biosensor for glyphosate based on the inhibition of tyrosinase conjugated to carbon nano-onions in a chitosan matrix on a screen-printed electrode. Microchim Acta 186:569 10.1007/s00604-019-3672-6 31338611

[5] Ding X, Yang K-L (2013) Development of an oligopeptide functionalized surface plasmon resonance biosensor for online detection of glyphosate. Anal Chem 85:5727–5733 23675691 10.1021/ac400273g

[6] Tarone RE (2018) On the international agency for research on cancer classification of glyphosate as a probable human carcinogen. Eur J Cancer Prev 27:82–87 27552246 10.1097/CEJ.0000000000000289

[7] Muñoz JP, Bleak TC, Calaf GM (2021) Glyphosate and the key characteristics of an endocrine disruptor: a review. Chemosphere 270:128619 33131751 10.1016/j.chemosphere.2020.128619

[8] Sanadgol N, Barati M, Houshmand F, Hassani S, Clarner T, Shahlaei M, Golab F (2020) Metformin accelerates myelin recovery and ameliorates behavioral deficits in the animal model of multiple sclerosis via adjustment of AMPK/Nrf2/mTOR signaling and maintenance of endogenous oligodendrogenesis during brain self-repairing period. Pharmacol Rep 72:641–658 32048246 10.1007/s43440-019-00019-8

[9] Koskinen WC, Marek LJ, Hall KE (2016) Analysis of glyphosate and aminomethylphosphonic acid in water, plant materials and soil. Pest Manag Sci 72:423–432 26454260 10.1002/ps.4172

[10] Masci M, Caproni R, Nevigato T (2024) Chromatographic methods for the determination of glyphosate in cereals together with a discussion of its occurrence, accumulation, fate, degradation, and regulatory status. Methods Protoc 7:38 38804332 10.3390/mps7030038 PMC11130892

[11] Hidalgo C, Rios C, Hidalgo M, Salvadó V, Sancho JV, Hernández F (2004) Improved coupled-column liquid chromatographic method for the determination of glyphosate and aminomethylphosphonic acid residues in environmental waters. J Chromatogr A 1035:153–157 15117086 10.1016/j.chroma.2004.02.044

[12] Valle A, Mello F, Alves-Balvedi R, Rodrigues L, Goulart L (2019) Glyphosate detection: methods, needs and challenges. Environ Chem Lett 17:291–317

[13] Emonds-Alt G, Malherbe C, Kasemiire A, Avohou HT, Hubert P, Ziemons E, Monbaliu J-C, Eppe G (2022) Development and validation of an integrated microfluidic device with an in-line Surface Enhanced Raman Spectroscopy (SERS) detection of glyphosate in drinking water. Talanta 249:123640 35716473 10.1016/j.talanta.2022.123640

[14] Yang X, Pang X, Sun L, Li W, Wang Y, Hua R, Zhu M (2024) A novel “Turn-Off-On” fluorescent probe for specific sequential detection of Cu2 + and glyphosate and its application in biological imaging. Spectrochim Acta A Mol Biomol Spectrosc 317:124420 38728848 10.1016/j.saa.2024.124420

[15] Aiolfi TR, Dagnino D, Schripsema J (2024) Holistic analysis of glyphosate formulations with nuclear magnetic resonance, similarity calculations and differential NMR. Microchem J 205:111199

[16] Ciasca B, Pecorelli I, Lepore L, Paoloni A, Catucci L, Pascale M, Lattanzio VMT (2020) Rapid and reliable detection of glyphosate in pome fruits, berries, pulses and cereals by flow injection–Mass spectrometry. Food Chem 310:125813 31757486 10.1016/j.foodchem.2019.125813

[17] Zambrano-Intriago LA, Amorim CG, Rodríguez-Díaz JM, Araújo AN, Montenegro MC (2021) Challenges in the design of electrochemical sensor for glyphosate-based on new materials and biological recognition. Sci Total Environ 793:148496 34182449 10.1016/j.scitotenv.2021.148496

[18] Buledi JA, Hyder A, Solangi AR, Shah Z-u-H, Darabi R, Karimi-Maleh H (2024) Nano-diamonds: transformative nanoscale material in advancing biosensor technology. Inorg Chem Commun 160:111934

[19] Hyder A, Memon SS, Memon S, Memon Z-u-A, Rajpar DB, Shaikh SG, Buledi JA (2021) A highly discerning p-tetranitrocalix [4] arene (p-TNC4) functionalized copper nanoparticles: a smart electrochemical sensor for the selective determination of diphenhydramine drug. Microchem J 163:105908

[20] Hyder A, Ali A, Buledi JA, Memon R, Al-Anzi BS, Memon AA, Kazi M, Solangi AR, Yang J, Thebo KH (2024) A NiO-nanostructure-based electrochemical sensor functionalized with supramolecular structures for the ultra-sensitive detection of the endocrine disruptor bisphenol S in an aquatic environment. Phys Chem Chem Phys 26:10940–10950 38526327 10.1039/d4cp00138a

[21] Solangi SA, Baig JA, Solangi IB, Afridi HI, Hussain S, Akhtar K, Khan LU, Abbasi F, Hyder A (2025) Bismuth ferrite polyaniline nanohybrid based electrochemical detection of theophylline. Mater Res Bull 188:113386

[22] Hyder A, Buledi JA, Memon AA, Ghanghro A, ur Rehman M, Thebo KH (2025) MXene-based nanocomposites: a new horizon for electrochemical monitoring of environmental pollutants. RSC Sustain 3:2160–2184

[23] Maghsoudi AS, Akmal MR, McClements DJ, Sani MA, Torabi R, Ataei M, Ganjali MR, Abdollahi M, Hassani S (2024) Determination of glyphosate using electrochemical aptamer-based label-free voltammetric biosensing platform. Microchem J 203:110897

[24] Kergaravat SV, Fabiano SN, Soutullo AR, Hernández SR (2021) Comparison of the performance analytical of two glyphosate electrochemical screening methods based on peroxidase enzyme inhibition. Microchem J 160:105654

[25] Omurlu B, Kizilkurtlu AA, Demirbas E (2025) Development of copper nanoparticles and multi-walled carbon nanotubes modified sensor system for effective and practical detection of glyphosate. Int J Environ Anal Chem. 10.1080/03067319.2025.2507839

[26] Mbokana JGY, Dedzo GK, Ngameni E (2020) Grafting of organophilic silane in the interlayer space of acid-treated smectite: application to the direct electrochemical detection of glyphosate. Appl Clay Sci 188:105513

[27] Fernandes JO, Bernardino CAR, dos Santos Fernandes J, Mahler CF, Braz BF, dos Santos LHC, Corrêa RJ, Santelli RE, Archanjo BS, Ribeiro ES (2023) Direct electrochemical determination of glyphosate herbicide using a screen-printed carbon electrode modified with carbon black and niobium nanoparticles. J Anal Test 7:425–434

[28] Qureashi A, Pandith AH, Bashir A, Malik LA, Manzoor T, Sheikh FA, Fatima K (2023) Z.-u. haq, electrochemical analysis of glyphosate using porous Biochar surface corrosive nZVI nanoparticles. Nanoscale Adv 5:742–755 36756521 10.1039/d2na00610c PMC9890542

[29] Moro G, Khaliha S, Pintus A, Mantovani S, Feltracco M, Gambaro A, Marforio TD, Calvaresi M, Palermo V, Melucci M (2024) Amino acid modified graphene oxide for the simultaneous capture and electrochemical detection of glyphosate. Mater Today Chem 36:101936

[30] Butmee P, Samphao A, Tumcharern G (2022) Reduced graphene oxide on silver nanoparticle layers-decorated titanium dioxide nanotube arrays as SERS-based sensor for glyphosate direct detection in environmental water and soil. J Hazard Mater 437:129344 35753303 10.1016/j.jhazmat.2022.129344

[31] Habekost A (2017) Rapid and sensitive spectroelectrochemical and electrochemical detection of glyphosate and AMPA with screen-printed electrodes. Talanta 162:583–588 27837875 10.1016/j.talanta.2016.10.074

[32] Zhang Q, Gu C, Singh R, Zhang B, Kumar S (2024) Development of waveflex biosensor for rapid detection of glyphosate herbicide in real agricultural products. IEEE Sens J 24:14320–14327

[33] Noori JS, Dimaki M, Mortensen J, Svendsen WE (2018) Detection of glyphosate in drinking water: a fast and direct detection method without sample pretreatment. Sensors 18:2961 30189680 10.3390/s18092961 PMC6163928

[34] Vidic J, Novakovic Z, Vasiljevic Z, Nikolić MV, Tadić NB, Djordjevic T, Radovic M, Gadjanski I, Papović S, Vlahović F (2025) ZnO-nanostructured electrochemical sensor for efficient detection of glyphosate in water, Available at SSRN 5084742

[35] Rowsell JL, Yaghi OM (2004) Metal–organic frameworks: a new class of porous materials. Microporous Mesoporous Mater 73:3–14

[36] Kitagawa S, Kitaura R, Noro Si (2004) Functional porous coordination polymers. Angew Chem Int Ed 43:2334–2375 10.1002/anie.200300610 15114565

[37] Zhang J, Tan Y, Song W-J (2020) Zeolitic imidazolate frameworks for use in electrochemical and optical chemical sensing and biosensing: a review. Microchim Acta 187:234 10.1007/s00604-020-4173-3 32180011

[38] Dong Y, Duan C, Sheng Q, Zheng J (2019) Preparation of Ag@ zeolitic imidazolate framework-67 at room temperature for electrochemical sensing of hydrogen peroxide. Analyst 144:521–529 30398238 10.1039/c8an01641k

[39] DMello ME, Sundaram NG, Kalidindi SB (2018) Assembly of ZIF-67 metal–organic framework over tin oxide nanoparticles for synergistic chemiresistive CO2 gas sensing. Chem Eur J 24:9220–9223 29722452 10.1002/chem.201800847

[40] Kim K, Kim J, Bae Y-S (2022) Zn–Co bimetallic zeolitic imidazolate frameworks as nonenzymatic electrochemical glucose sensors with enhanced sensitivity and chemical stability. ACS Sustain Chem Eng 10:11702–11709

[41] Yang N, Zhou X, Qi X, Li J, Fang W, Xue H, Yang Z (2022) A nitrite sensor based on bimetallic zeolitic imidazole framework derived Co/porous carbon nanorods. Microchem J 182:107910

[42] Elfiky M, Salahuddin N, Matsuda A (2020) Green fabrication of 3D hierarchical blossom-like hybrid of peeled montmorillonite-ZnO for in-vitro electrochemical sensing of diltiazem hydrochloride drug. Materials Science and Engineering: C 111:110773 32279745 10.1016/j.msec.2020.110773

[43] Alizadeh A, Asghar S, Roudgar-Amoli M, Shariatinia Z, Protection E (2023) Water remediation using activated montmorillonite/metal-organic framework nanocomposites: response surface methodology, kinetic, and thermodynamic studies. 177:507–529

[44] Elfiky M, Abdo M, Darwesh M, Salahuddin N (2025) Ultra-sensitive detection of 4-chloro-2-methylphenoxyacetic acid herbicide using a porous Co-1, 4-benzenedicarboxylate/montmorillonite nanocomposite sensor. Microchim Acta 192:30 10.1007/s00604-024-06765-8 PMC11668838 39718606

[45] Elfiky M, Kumar R, Beltagi A (2022) Anthropogenic greenhouse CO_2_ gas sensor based on glassy carbon modified with organoclay/polypyrrole-alginate nanocomposites in brackish water and seawater. J Electroanal Chem 926:116926

[46] Elfiky M, Beltagi AM, Abuzalat O (2024) Adsorptive stripping voltammetric sensor based on Cd zeolitic imidazole framework-67 for electrochemical detection of sarin simulant. Microchim Acta 191:80 10.1007/s00604-023-06112-3 PMC10774163 38190052

[47] Nguyen TN, Nguyen HP, Kim T-H, Lee SW (2018) Bimetallic Co/Zn-ZIF as an efficient photocatalyst for degradation of Indigo carmine. Korean J Mater Res 28:68–74

[48] Elfiky M, Ghoneim M, El-Desoky H, Hassanein A, Salahuddin N (2023) Electrochemical stripping voltammetrical sensor based on polypyrrole exfoliated polyetheramine–montmorillonite nanocomposite for nanomolar detection of Nifuroxazide. RSC Adv 13:5107–5117 36777946 10.1039/d2ra06160k PMC9909373

[49] Jia M, Zhang Z, Wei L, Li J, Yuan D, Wu X, Mao Z (2019) High-and low-temperature properties of layered silicate-modified bitumens: view from the nature of pristine layered silicate. Appl Sci 9:3563

[50] Murisi MA, Al-Asheh S, Abdelkareem MA, Aidan A, Elsaid K, Olabi AG (2023) In situ growth of zeolite imidazole frameworks (ZIF-67) on carbon cloth for the application of oxygen reduction reactions and microbial fuel cells. ACS Omega 8:44514–44522 38046312 10.1021/acsomega.3c02544 PMC10688201

[51] Habibi B, Bahadori Y, Pashazadeh S, Pashazadeh A (2025) ZIF-67 decorated with silica nanoparticles and graphene oxide nanosheets composite modified electrode for simultaneous determination of paracetamol and diclofenac. Sci Rep 15:9499 40108259 10.1038/s41598-025-94178-9 PMC11923285

[52] Habibi B, Pashazadeh S, Saghatforoush LA, Pashazadeh A (2021) A thioridazine hydrochloride electrochemical sensor based on zeolitic imidazolate framework-67-functionalized bio-mobile crystalline material-41 carbon quantum Dots. New J Chem 45:14739–14750

[53] Xie M, Yang D, Yang C, Ning R, Xu H, Wang J (2024) Comparison of ZIFs and MWCNTs co-optimization on the dielectric properties and thermal conductivity of epoxy-based composites. J Inorg Organomet Polym Mater 34:3473–3482

[54] Bard AJ, Faulkner LR, White HS (2022) Electrochemical methods: fundamentals and applications. John Wiley & Sons

[55] Feng D, Soric A, Boutin O (2020) Treatment technologies and degradation pathways of glyphosate: a critical review. Sci Total Environ 742:140559 32629265 10.1016/j.scitotenv.2020.140559

[56] Heller-Kallai L, Bergaya F, Theng B, Lagaly G (2006) Handbook of clay science

[57] Elfiky M, Abdo Mm, Darwesh M, Salahuddin N (2025) Ultra-sensitive detection of 4-chloro-2-methylphenoxyacetic acid herbicide using a porous Co-1, 4-benzenedicarboxylate/montmorillonite nanocomposite sensor. Microchim Acta 192:1–14 10.1007/s00604-024-06765-8 PMC11668838 39718606

[58] Erk N, Kurtay G, Bouali W, Sakal Z, Genç A, Erbaş Z, Soylak M (2024) Electrochemical detection of melphalan in biological fluids using a g-C3N4@ ND-COOH@ MoSe_2_ modified electrode complemented by molecular docking studies with cellular tumor antigen P53. ACS Omega 9:21058–21070 38764632 10.1021/acsomega.4c00558 PMC11097377

[59] Laviron E, Roullier L, Degrand C (1980) A multilayer model for the study of space distributed redox modified electrodes: part II. Theory and application of linear potential sweep voltammetry for a simple reaction. J Electroanal Chem Interfacial Electrochem 112:11–23

[60] Treviño-Reséndez J, Soto-Hernández E, Godínez LA, Robles I, Meas Vong Y, García-Espinoza JD (2024) Electrochemical oxidation of glyphosate using graphite rod electrodes: impact of acetic acid pretreatment on degradation efficiency. Processes 12:2359

[61] Sen K, Datta JK, Mondal NK (2021) Box–Behnken optimization of glyphosate adsorption on to biofabricated calcium hydroxyapatite: kinetic, isotherm, thermodynamic studies. Appl Nanosci 11:687–697

[62] Chen L, Li P, Li K, Zhao S, Chen M, Pan W, Liu Y, Li Z (2025) Zeolite imidazole frame-67 (ZIF-67) and its derivatives for pollutant removal in water: a review. Processes 13:1724

[63] Wong A, de Lima DG, Ferreira PA, Khan S, da Silva RAB, de Faria JLB, Del Pilar Taboada Sotomayor M (2021) Voltammetric sensing of glyphosate in different samples using carbon paste electrode modified with biochar and copper (II) hexadecafluoro-29H, 31 phtalocyanine complex. J Appl Electrochem 51:761–768

[64] Anbari AP, Delcheh SR, Kashif M, Ranjbari A, Karbalaei Akbari M, Zhuiykov S, Heynderickx PM, Verpoort F (2025) Engineering Fe-modified zeolitic imidazolate frameworks (Fe-ZIF-8 and Fe-ZIF-67) via in situ thermal synthesis for enhanced adsorption of malachite green from aqueous solutions: a comprehensive study of isotherms, kinetics, and thermodynamics. Nanomaterials 15:1097 40711216 10.3390/nano15141097 PMC12298564

[65] Giacomazzo GE, Paderni D, Giorgi L, Formica M, Mari L, Montis R, Conti L, Macedi E, Valtancoli B, Giorgi C (2023) A new family of macrocyclic polyamino biphenolic ligands: acid-base study, Zn (II) coordination and glyphosate/AMPA binding. Molecules 28:2031 36903278 10.3390/molecules28052031 PMC10003900

[66] Traiwatcharanon P, Velmurugan S, Zacharias M, Wongchoosuk C (2023) Sparked ZnO nanoparticles-based electrochemical sensor for onsite determination of glyphosate residues. Nanotechnology 34:415501 10.1088/1361-6528/ace3cc 37402361

[67] Beitollahi H, Dourandish Z, Tajik S, Jahani PM (2025) Electrochemical Sensors in Agriculture. Agricultural Electrochemistry. ACS Publications, pp 163–179

[68] Lopes BV, Maron GK, Masteghin MG, Balboni RDC, Silva SRP, Carreno NLV (2025) Direct-detection of glyphosate in drinking water via a scalable and low-cost laser-induced graphene sensor. Anal Methods 17:808–815 39713940 10.1039/d4ay01549e

[69] Zúñiga K, Rebollar G, Avelar M, Campos-Terán J, Torres E (2022) Nanomaterial-based sensors for the detection of glyphosate. Water 14:2436

[70] Yuliani T, Saepudin E, Ivandini T (2018) Anodic stripping voltammetry of Ni (OH) 2 nanoparticles in acid solution using boron-doped diamond electrodes, AIP Conference Proceedings, AIP Publishing LLC, 020096

[71] Franz JE, Mao MK, Sikorski JA (1997) Glyphosate: a unique global herbicide

[72] Pintado S, Amaro RR, Mayén M, Mellado JMR (2012) Electrochemical determination of the glyphosate metabolite aminomethylphosphonic acid (AMPA) in drinking waters with an electrodeposited copper electrode. Int J Electrochem Sci 7:305–312

[73] Tran N, Drogui P, Doan TL, Le TS, Nguyen HC (2017) Electrochemical degradation and mineralization of glyphosate herbicide. Environ Technol 38:2939–2948 28112035 10.1080/09593330.2017.1284268

