# Supplementary material for: Ultra-sensitive glyphosate detection in soil and wastewater using Zn-zeolitic imidazolate framework-67/montmorillonite nanocomposite electrochemical sensor
Source: Mikrochim Acta. 2026 Mar 11;193(4):232. doi: 10.1007/s00604-026-07880-4 (PMC12975828; doi:10.1007/s00604-026-07880-4)
Supplement: Supplementary file 1 — (DOCX 344 KB) [file 604_2026_7880_MOESM1_ESM.docx]

**Ultra-Sensitive Glyphosate Detection in Soil and Wastewater Using Zn-Zeolitic Imidazolate Framework-67 /Montmorillonite Nanocomposite Electrochemical Sensor**

Mona Elfiky ^a^*, Amr. M. Beltagi^b^, Marwa M. Bediwy ^a^

^a^Chemistry Department, Faculty of Science, Tanta University, Tanta, 31527 Egypt

^b^Department of Chemistry, Faculty of Science, Kafrelsheikh University, 33516 Kafrelsheikh, Egypt

^*^Corresponding author e-mail: [Elfiky_mona@science.tanta.edu.eg](mailto:Elfiky_mona@science.tanta.eu.eg&osama.abuzalat@mtc.edu.eg)


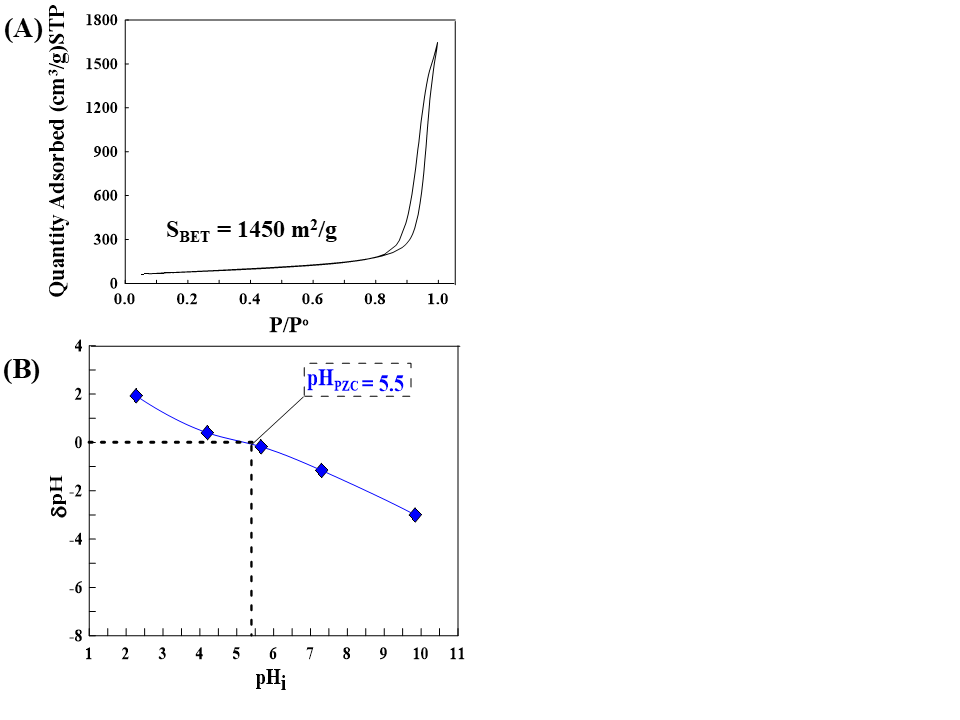


**Figure S_1_: (A)** N_2_ adsorption-desorption isotherms of Zn-ZIF-67/0.5 Exf. MMt framework, (B) its corresponding pore size distribution. **(B)** The plot of ***δpH*** (***pH*_f_**-***pH*_i_**) vs. ***pH*_i_** including the value of ***pH*_ZPC_** of Zn-ZIF-67/0.5 Exf.MMt framework.


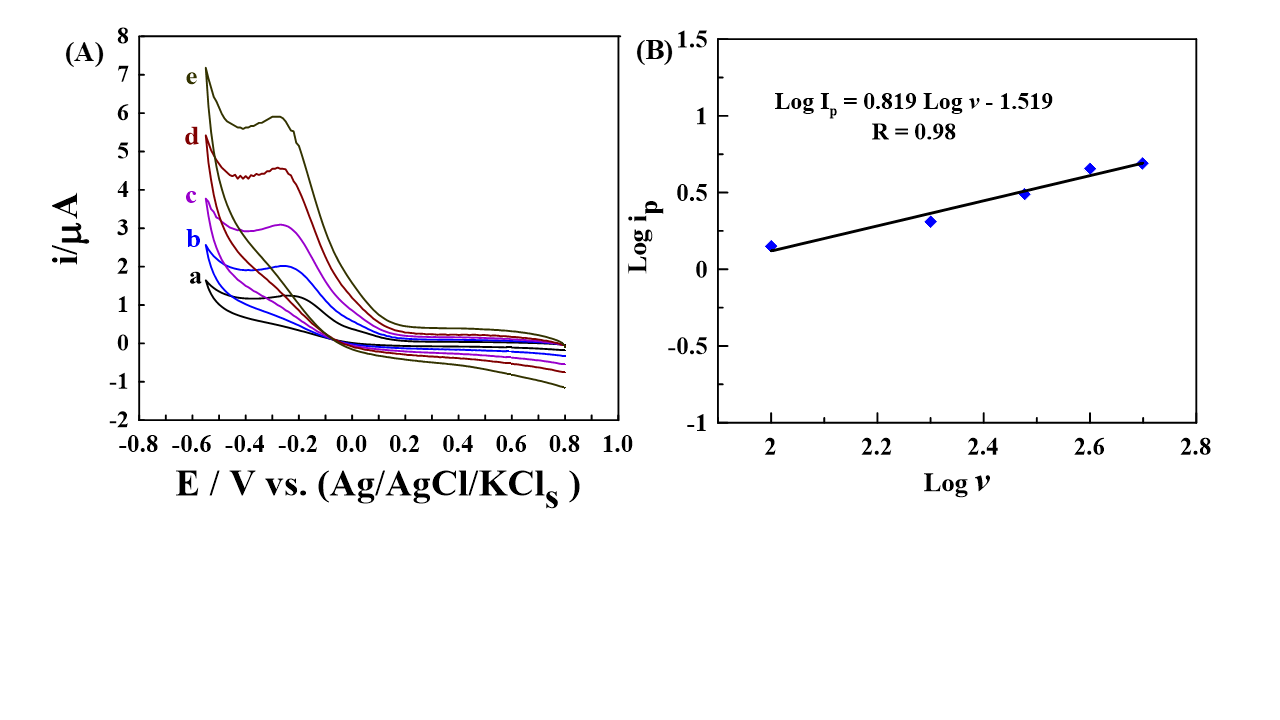


**Figure S_2_: (A)** CVs of 0.1 nM GLY in 0.1 M HCl at 100 mV·s^-1^, and **(B)** log *I*_p_ vs. log ν plot from CVs of 0.1 nM GLY at scan rates of 100–500 mV·s^-1^ using 1.0% [Zn-ZIF-67/2.0 Exf. MMt] MGPS.


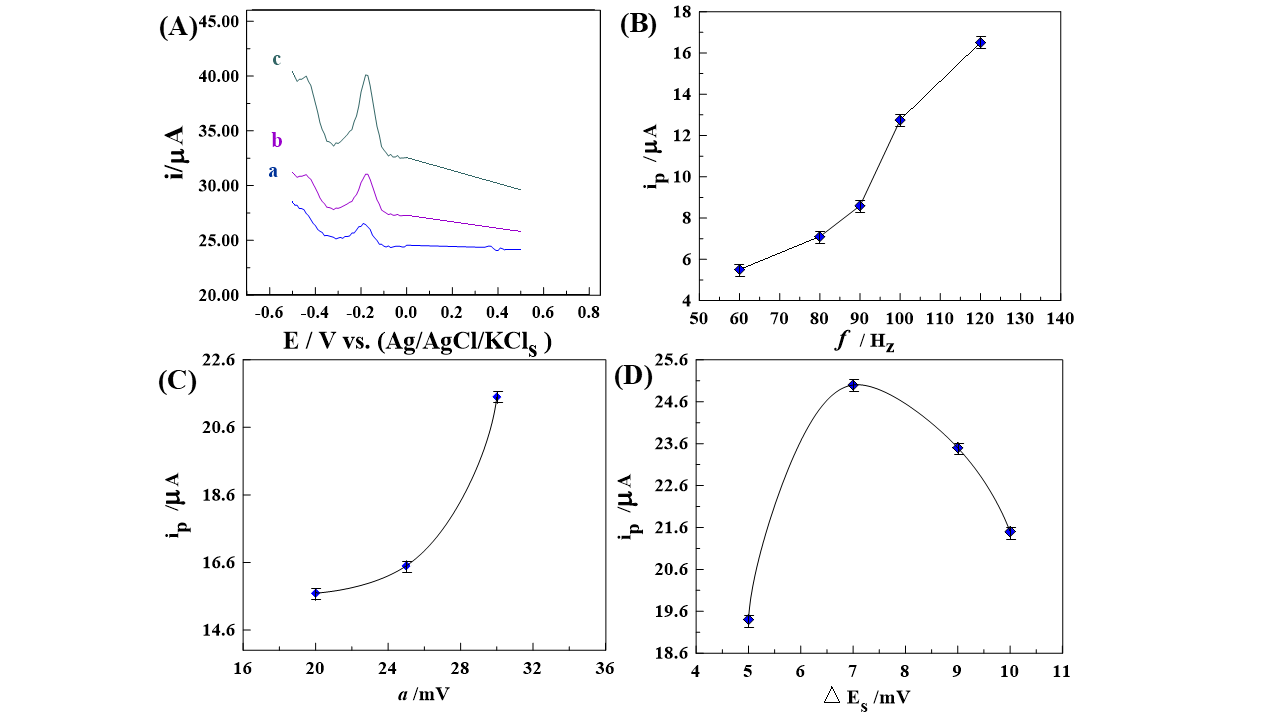


**Figure S_3_: (A)** Effect of changing type of supporting electrolyte **(a)** BRB (pH 2), **(b)** PBS (pH 2), (c) 0.1 M HCl. Influence of **(B)** frequency (*f*), **(C)** pulse amplitude (*a*), and (**D)** scan increment (∆*E_s_*) upon the surface of the 1.0 % [Zn-ZIF-67/0.5 Exf. MMt] MGPS at *E_acc_* = 0.5 V for 100 s.


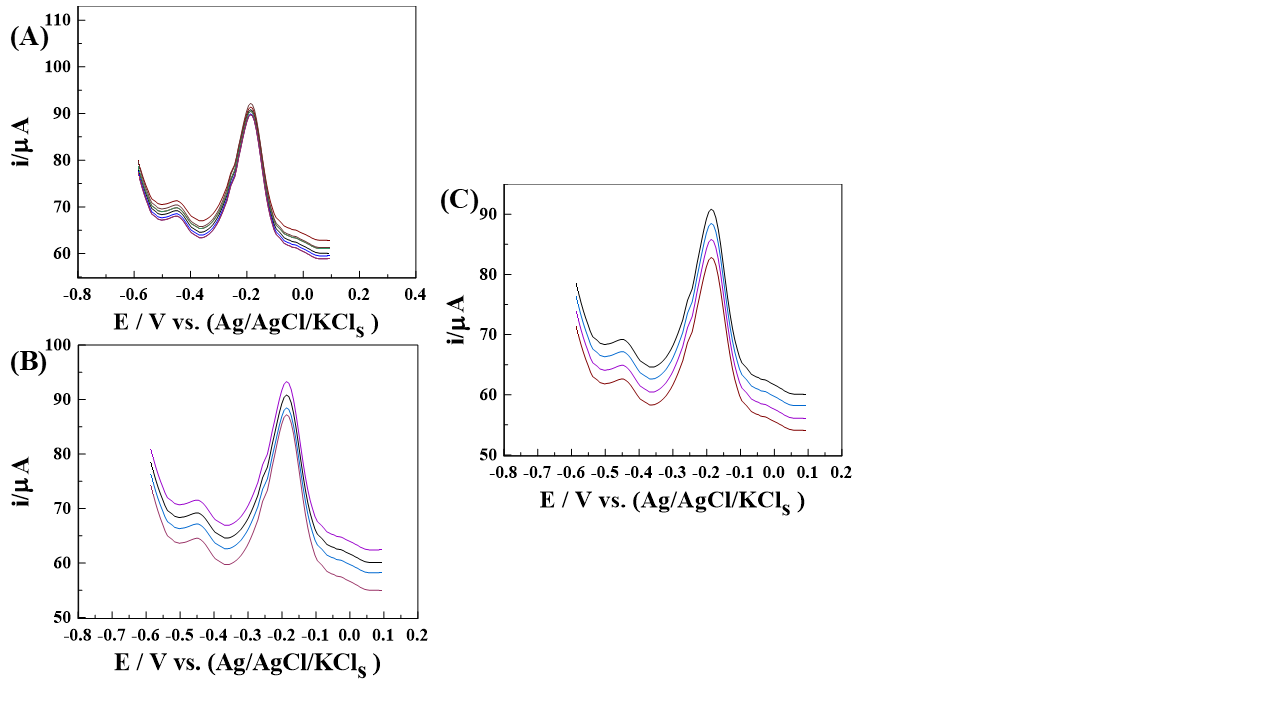


**Figure S_4_:** Histogram of **(A)** intra-day precision, **(B)** repeatability, and **(C)** stability of 0.09 nM GLY in 0.1 M HCl on the surface of 1.0% [Zn-ZIF-67/0.5 Exf. MMt] MGPS.
